# Traditional Therapeutics and Potential Epidrugs for CVD: Why Not Both?

**DOI:** 10.3390/life14010023

**Published:** 2023-12-22

**Authors:** Lauren Rae Gladwell, Chidinma Ahiarah, Shireen Rasheed, Shaikh Mizanoor Rahman, Mahua Choudhury

**Affiliations:** 1 Department of Pharmaceutical Sciences, Texas A&M Irma Lerma Rangel College of Pharmacy, 1114 TAMU, College Station, TX 77843, USA; 2 Natural and Medical Sciences Research Center, University of Nizwa, Birkat Al-Mouz, Nizwa 616, Oman

**Keywords:** cardiovascular diseases (CVDs), epigenetics, CVD treatment, traditional CVD medications, epigenetic CVD medications, epidrugs, statins, calcium channel blockers, beta blockers

## Abstract

Cardiovascular disease (CVD) is the leading cause of death worldwide. In addition to the high mortality rate, people suffering from CVD often endure difficulties with physical activities and productivity that significantly affect their quality of life. The high prevalence of debilitating risk factors such as obesity, type 2 diabetes mellitus, smoking, hypertension, and hyperlipidemia only predicts a bleak future. Current traditional CVD interventions offer temporary respite; however, they compound the severe economic strain of health-related expenditures. Furthermore, these therapeutics can be prescribed indefinitely. Recent advances in the field of epigenetics have generated new treatment options by confronting CVD at an epigenetic level. This involves modulating gene expression by altering the organization of our genome rather than altering the DNA sequence itself. Epigenetic changes are heritable, reversible, and influenced by environmental factors such as medications. As CVD is physiologically and pathologically diverse in nature, epigenetic interventions can offer a ray of hope to replace or be combined with traditional therapeutics to provide the prospect of addressing more than just the symptoms of CVD. This review discusses various risk factors contributing to CVD, perspectives of current traditional medications in practice, and a focus on potential epigenetic therapeutics to be used as alternatives.

## 1. Introduction

Cardiovascular disease (CVD) is a major threat to health worldwide, as the leading cause of death [1]. In the United States alone, CVD is responsible for causing one in every five deaths [2]. CVD encompasses but is not limited to vascular diseases, peripheral artery disease, stroke, hypertension, and atherosclerosis as well as various heart diseases such as heart failure and coronary heart disease [3]. As of 2019, specifically ischemic heart disease and ischemic stroke are responsible for being the leading causes of CVD death in the United States [4]. The debilitating toll that CVD takes on human life is only made heavier by the cost of patient care. Effective treatment for these diseases is a huge economic burden, with an estimated cost of over USD 400 billion being spent in the United States just from 2018 to 2019 [5]. Graver is that the surging of the global dual epidemics of obesity and diabetes that can contribute to the development of CVD [6,7,8]. This paints a daunting future for health worldwide if left unaddressed.

Astonishingly, in the past almost 30 years, CVD incidence and mortality rates have dramatically decreased [9]. This decline can be attributed to the fact that many preventative measures were introduced at the turn of the century [10]. Efforts to promote healthy behaviors as a means for prevention included public smoking bans, the accurate monitoring of blood pressure, as well as the implementation of medications to control low-density lipoprotein (LDL) cholesterol or cardio-protective medications such as aspirin [10]. Recently, the American Heart Association (AHA) has introduced the updated “Life’s Essential 8” to help mitigate the overwhelming burden of CVD by providing an enhanced system for the measurement of cardiovascular health [11]. This system can guide both clinical recommendations and patient decisions towards achieving better cardiovascular health to prevent and treat CVD. Disparities with this system, however, still exist pertaining to race, ethnicity, age, socioeconomic standing, and other characteristics [12,13]. These multiple factors can contribute to why, despite prior initiatives, CVD still maintains its status as the leading cause of death worldwide [1]. These circumstances thus demand an arduous shift from conventional therapeutic approaches to overcome this crisis.

Epigenetics is a branch of science that refers to the ability of the genome to adapt to the environment through the modulation of its organization. By altering factors surrounding DNA, such as the organization of chromatin or the methylation of DNA, there can thus be the regulation of gene expression [14]. Gene expression can be controlled by several external factors including prenatal malnutrition, ultraviolet radiation, plasticizer exposure, smoking, etc. For example, a poor diet and perinatal nutrition can cause deviations in DNA methylation, which can make an individual more susceptible to metabolic diseases [15]. The DNA methylation of specific genes can prevent their expression, while variations in DNA methylation can result in phenotypic effects such as changes in body weight and blood pressure [16,17]. Recent studies also suggest that parental habits and environmental exposures can also affect one’s offspring’s epigenome; therefore, the inheritance of altered gene expression can highly influence a person’s risk of CVD [14,18]. Thus, epigenetic drugs, also known as epidrugs, present a potential alternative to traditional therapeutics by offering the benefit of addressing epigenetic insults incurred throughout life and even those inherited transgenerationally. This review discusses potential targets for CVD therapeutics through contributing risk factors, current medications that are implemented in traditional practice, and a comparison with novel epidrug candidates for the treatment of CVD.

## 2. CVD Risk Factors

### 2.1. Obesity

Obesity is defined as the excessive accumulation of adipose tissue. A person is considered overweight if their body mass index (BMI) ranges between 25 kg/m^2^ and 29.9 kg/m^2^, obese if their BMI is greater than or equal to 30 kg/m^2^, and severely obese if their BMI is greater than or equal to 40 kg/m^2^ [19]. By 2030, it is predicted that almost half of the United States will be obese [20]. More dire is that over the past approximately 50 years, the prevalence of severe obesity has increased almost ten-fold [21]. Obesity is a key risk factor for CVD, as the accumulation of body fat can incite a vicious cycle leading to the deterioration of metabolic health and the promotion of disease development.

To compensate for the excess lipids that are present due to overnutrition, characteristic of obesity, adipose cells must undergo remodeling. This involves increasing the size and the number of adipose cells in the body; however, this remodeling processes induces stress on the cells [22]. In response to stress, the cells will release several proinflammatory adipokines and cytokines, thereby directly increasing inflammation. There can also be an allocation of lipids to other tissues, such as to the liver or within blood vessels, if the adipose cells are unable to keep up with the influx of lipids [23]. This chronic inflammatory environment and excess lipid content set the stage for the development of CVD. Several studies have exhibited that diet and exercise can reduce obesity and the risk of CVD-related morbidity; however, these interventions may not be maintainable [24]. Therefore, perhaps a new approach involving epigenetics for targeting obesity can potentially provide therapeutic value in combating CVD.

Recent evidence has demonstrated that several epigenetic mechanisms can contribute to the development of obesity and CVD. The methylation of the promoter region for the hormone leptin, which is responsible for eliciting a sensation of fullness, was found to have an inverse correlation with body weight, where reduced promoter methylation was observed in obese participants [25]. Typically, the hypomethylation of promoter regions is indicative of gene upregulation. This can contribute to the explanation of how there is a compensatory upregulation of leptin in obesity that then results in leptin resistance [26]. Another example is demonstrated by Mikula et al., where there was increased histone acetylation on histone 3 lysine 9 and 18 of proinflammatory genes in mice fed on a high-fat diet [27]. These increased activating histone marks can thereby contribute to the elevation of inflammation associated with obesity. This promotion of inflammation can also exacerbate the risk of CVD development, making these histone modifications worthy therapeutic targets. Additionally, increased acetylated lysine and B-type natriuretic peptide (BNP) was observed alongside downregulated Sirtuin (SIRT) 3 expression in cardiac tissue from obese patients with heart failure [28]. The downregulation of SIRT3 was proposed to induce mitochondrial dysfunction through increased membrane permeability via the hyperacetylation of cyclophilin D. A similar scenario was described by Romanick et al., where the enlarged hearts of obese mice displayed increased BNP and lysine acetylation [29]. Further analysis revealed that pathways relating to mitochondrial dysfunction, oxidative phosphorylation, calcium signaling, and SIRT signaling were dysregulated by obesity. By evaluating overlapping therapeutic targets, the reversible nature of epigenetics can be leveraged to combat both obesity and CVDs.

### 2.2. Type 2 Diabetes Mellitus (T2DM)

Currently, there are 537 million diabetic adults worldwide, with the vast majority suffering from T2DM [30]. By 2030, it is projected that the global prevalence will increase to 643 million, and by 2045, it will reach 783 million. Diabetes places an overwhelming financial burden on the United States healthcare system. As of 2017 an estimated USD 327 billion was spent on diabetes-related medical costs [31]. It is established that T2DM is a significant risk factor for CVD; people with T2DM can experience a two- to fourfold increase in cardiovascular risk compared to those without diabetes [32]. Hyperglycemia that is characteristic of T2DM can specifically accelerate the development of atherosclerosis and thereby Atherosclerotic Cardiovascular Disease (ASCVD) [33]. Uncontrolled hyperglycemia, another feature of T2DM, causes extensive endothelial damage and elicits inflammatory responses mediated by macrophage release, thereby exacerbating plaque buildup and atherosclerosis [34,35]. Epidemiological studies indicate that improvements in T2DM management have led to significant reductions in diabetes-associated cardiovascular morbidity [36]. More recently, a new class of antidiabetic pharmaceuticals known as sodium glucose co-transporter (SGLT2) inhibitors have exhibited promise in ameliorating T2DM symptoms as well as reducing cardiovascular complications [37]. These preliminary improvements can potentially be further propelled if epigenetics is also considered.

Several epigenetic modifications such as DNA methylation, histone modification, and noncoding RNAs have been associated with the prognosis of T2DM [38,39]. As epigenetics bridges our genome and the environment, there are environmental factors that can similarly promote both CVD and T2DM disease progression. These factors can then lead to consequences that resonate from within cells and propagate throughout the entire body [40]. A hyperglycemic environment inhibited the protective JunD proto-oncogene subunit (JunD) expression in diabetic mouse model hearts, later validated in the hearts of T2DM patients, and was proposed to be mediated by several epigenetic mechanisms [41]. Specifically, diabetic mice exhibited the hypermethylation of two regions of CpG islands, decreased promoter histone 3 lysine 4 mono- and trimethylation, increased promoter histone 3 lysine 9 trimethylation, and reduced microRNA-673 expression. This emphasizes the overlapping layers of epigenetic regulation that can interact to direct gene expression. Further, diminished microRNA-24 expression was found to be exacerbated in patients with coronary heart disease (CHD) and T2DM compared to patients with only CHD or neither diagnosis [42]. This was accompanied by an inversely proportionate increase in chitinase 3-like 1 (YKL-40) that was found to be a downstream target of microRNA-24 and negatively correlated with its expression in either circumstance of patients with CHD and T2DM or CHD alone. Upregulated YKL-40 was exhibited in several inflammatory diseases, especially T2DM and coronary artery disease, and highlights how proinflammatory processes can contribute to disease development [43]. For instance, increased cellular oxidative stress, endoplasmic reticulum (ER) stress, and mitochondrial dysfunction can mediate inflammation that exacerbates endothelial dysfunction and insulin resistance [44,45]. By targeting the epigenetic dysregulations incurred, we can address more than just the symptoms of T2DM and CVD.

### 2.3. Smoking

In 2018, an estimated 13.7% (34.2 million) of U.S. adults were cigarette smokers [46]. Smoking is well established in causing vascular dysfunction through reduced nitric oxide bioavailability, the increased expression of adhesion molecules, increased inflammation, the activation of prothrombic factors, and the promotion of endothelial dysfunction [47]. Cigarette smoke contains toxic chemicals and carcinogens whose oxidants and free radicals create a pro-oxidative environment [48]. Inflammatory processes begin to ignite as macrophages take up these oxidative species and lipids to form foam cells that play a primary role in arterial lipid deposition and plaque formation [49]. Additionally, smoking is known to increase total serum cholesterol, VLDL, LDL, and triglyceride serum concentrations, which in turn increases the risk of atherosclerosis and other forms of CVDs [50]. The cessation of smoking was found to significantly reduce the risk of CVD events such as nonfatal myocardial infarctions, nonfatal strokes, and death [51]. The repercussions of smoking also do not just end at the individual level.

Increasing evidence suggests that maternal smoking can affect the offspring’s risk for developing CVD risk factors [52,53]. One study conducted by Power et al. found that individuals whose mothers smoked during pregnancy had a higher prevalence of metabolic syndrome in addition to increased BMI, waist circumference, blood pressure, HbA1c, and triglyceride levels on average compared to the offspring of non-smoking mothers [52]. This exemplifies an epigenetic effect of smoking on offspring from the actions of their mothers. Further, smoking can modulate interferon-gamma (INF-γ) transcription via histone acetyltransferases to contribute to proinflammatory effects and macrophage activation [54]. This is significant, as INF-γ is implicated in the priming of enhancers used to mediate the activation of inflammatory pathways and alter macrophage expression in the pathogenesis of CVD [54]. The promotion of apoptosis is another consequence of smoking demonstrated using nicotine-exposed cardiomyocytes, continuing to warn against the repercussions of smoking on cardiovascular tissue [55]. Apoptosis was theorized to be mediated by inhibited extracellular signal-regulated kinase (ERK) signaling leading to reduced serum response factor expression, decreased microRNA-133, and a subsequent increased expression of caspase-9 and caspase-3. This illustrates how disturbed epigenetic regulators such as microRNAs can act as both a receiver and inducer of CVD. Recently, dysregulated epigenetic modifications obtained during smoking were discovered to respond to cessation [56]. While some modifications can be reversed by cessation, there may be others that require further intervention. By considering epidrugs, there is the potential for ameliorating the damage caused by smoking as well as CVD development and progression.

### 2.4. Hypertension

The American College of Cardiology and the American Heart Association (ACC/AHA) define hypertension as a blood pressure level > 130/80 mmHg, and it afflicts 48% of the American population [57]. In addition to its high prevalence, hypertension adds a significant financial strain on the American health system, with an estimated USD 131 billion per year being spent on hypertension-related care [58]. Further, only half of Americans diagnosed with hypertension achieve adequate blood pressure control [59]. There also exists a racial disparity in this context, as a majority of minority groups are observed to have worsened control over their hypertension compared to non-Hispanic White individuals [60]. This causes major concern, as uncontrolled hypertension is a high-risk factor for CVD development. The continual pressure and strain on the blood vessels can cause them to narrow, weaken, and potentially burst, leading to an increased workload on the heart [61].

While there are several different pharmacological classes of anti-hypertensive medications, not all classes are equally effective across patients. For example, angiotensin-converting enzyme inhibitors (ACEIs) have displayed reduced efficacy in the Black community [62]. This characteristic should subsequently direct physicians’ decisions to not prescribe ACEIs as a first-line monotherapy for Black patients. Instead, a thiazide diuretic, calcium channel blocker, or combinatorial therapy should be considered [63]. While the precise mechanisms responsible have yet to be fully elucidated, there is evidence that suggests genetic and environmentally acquired contributions can play a role in this variable response [64]. Epigenetics can thus offer potential in discerning the impact between these two elements. In a recent epigenome-wide association study conducted on sub-Saharan African participants, van der Linden and colleagues were able to identify differentially methylated positions in genes such as Protein Tyrosine Phosphatase Receptor Type N2 (PTPRN2) that contribute to the homeostasis of the renin–angiotensin–aldosterone system [65]. Further, differing levels of methylation in genes, including PTPRN2, correlated with coronary heart disease and metabolic syndrome and were discovered to be specific to race [66,67]. Epigenetic modifications that play a role in blood pressure regulation can be targeted to overcome the barriers posed by traditional pharmaceutical therapeutics [68,69]. By also preceding the development of disease, epigenetics can also provide novel therapeutic targets. A study investigating potential biomarkers for hypertension identified microRNA-126, -221, and -222 to be inversely correlated with a rise in systolic blood pressure in a Japanese cohort across 5 years [70]. MicroRNAs (miRNA) can act as both enhancers or suppressors of gene expression and can be an early indicator of disease before the onset of symptoms. Our lab demonstrated the prowess of the miRNA biomarker miR-17 alongside other epigenetic modulators in the hypertensive pregnancy disorder of preeclampsia (PE), awarding us a patent [71]. Women that suffered from PE during pregnancy are twice as likely to develop CVD, bringing forth the magnanimous potential value that early intervention afforded by miRNAs can pose in both PE and CVD [72]. Collectively, this points to how epigenetics can provide prospective opportunities of addressing CVD at early time points, prior to the onset of symptoms.

### 2.5. Hyperlipidemia/Atherosclerosis

Hyperlipidemia is a condition associated with abnormal levels of lipids in the body that can be in the form of different kinds of cholesterol and/or triglycerides [73]. Due to its high occurrence, antihyperlipidemic pharmaceuticals are one of the most frequently prescribed medication classes in the United States [74]. Adults between the ages of 40 and 75 that exhibit at least one risk factor for CVD and that are at a 10% or greater risk for having a cardiovascular event in the next 10 years are recommended for statin therapy [75]. With the high prevalence of risk factors such as obesity, T2DM, smoking, and hypertension, there is an ongoing demand for aggressive lipid-lowering therapies [76].

A role by which hyperlipidemia is a major risk factor for CVD is that, if left unattenuated, hyperlipidemia can be conducive of atherosclerosis development [77,78]. Prolonged, increased lipid levels in the blood can lead to their accumulation along the arterial walls, resulting in the formation of plaques. These plaques can then contribute to inflammation as well as narrow and stiffen arterial walls, which defines atherosclerosis [79]. Epigenetic modifications have also been identified to participate in this process. In atherosclerotic lesions, there was a decreased methylation and increased acetylation of histone 3 lysine 9 and lysine 27 in smooth muscle cells taken from human carotid vessels [80]. Recently, DNA methyltransferase 3b (DNMT3b) was implicated in atherosclerosis progression, as its inhibition was found to ameliorate the plaque content, regulatory T cell (Treg) populations, and inflammation in apolipoprotein E (ApoE) knockout mice [81]. DNMT3b was described to be responsible for the hypermethylation of the regulatory T-cell-specific demethylated region of forkhead box P3 (FOXP3), resulting in decreased FOXP3 expression that in turn downregulated Treg levels. As Tregs are proposed to be protective due to their secretion of anti-inflammatory cytokines, decreased Treg populations can potentially contribute to the propagation of plaques and ultimately the pathogenesis of atherosclerosis [82]. Most concerning is that these plaques can ultimately lead to death. Plaques can rupture, causing subsequent injuries to the arteries, potentially resulting in acute thrombosis, and triggering acute coronary syndromes [83]. Thus, ASCVD was termed to describe the associated outcomes such as aortic aneurysm, aortic stenosis, peripheral arterial disease, coronary artery disease (CAD), non-fatal myocardial infarction (MI), stroke, and even sudden cardiac death [84].

Current guidelines for treatment include an assessment of cardiovascular risk in 10 years using the Framingham Risk Score (FRS) algorithm, which takes into consideration various factors such as LDL, age, race, gender, diagnosis of T2DM, systolic blood pressure, and smoker status [85,86]. While the overall incidence of ASCVD has declined over the course of many years, there still lies therapeutic disparities. Women, for instance, are observed to not only have poorer patient outcomes related to ASCVD but are also not equally offered preventative therapies (e.g., statins and antiplatelet therapies) compared to men [87,88,89]. While hyperlipidemia and atherosclerosis are known to be directly linked to CAD, the underpinning genetic regulations that contribute to these conditions remain elusive [90,91]. There are currently fewer than 200 genes that have been associated with hyperlipidemia, atherosclerosis, or CAD [92,93,94]. A few human studies have provided a promising link of differential gene expression in hyperlipidemia with inflammation and CAD, although there are no current drug therapies targeting these genes such as pro-platelet basic protein (PPBP) and α-defensin (DEFA1/DEFA3) [95]. This demonstrates the demand for more innovative therapeutic approaches.

## 3. Epigenetics

Epigenetics is the study of changes in gene expression without any alteration to the DNA sequence. This involves altering the organizational status of the chromatin. For example, modifications to the tails of histones such as acetylation can activate gene expression by loosening the chromatin structure [96]. The three key epigenetic modifications include histone modification, DNA methylation, and noncoding RNAs. Environmental factors such as diet and exposure to toxins can contribute to the regulation of these modifications that lead to modulated gene expression. When gene expression is dysregulated due to these affected epigenetic modifications, it can often lead to comorbidities such as metabolic disease [97]. Further, these modifications are heritable, which can result in the accumulation of altered epigenetic modifications across generations [98].

This phenomenon of a transgenerational effect has been demonstrated in evolution and adaptation. For example, Gonzalez et al. tested the effects of genome-wide DNA methylation on plants based on maternal stresses (drought, soil contaminations, and shading) [99]. Plants grown in copper-contaminated soils in the maternal generation demonstrated a positive transgenerational effect due to its importance in plant development [59]. In another study, two subsequent generations of offspring from rats fed on a high-fat diet experienced poor metabolic health such as higher blood glucose levels and decreased insulin secretion [100]. History has also provided examples where populations acquired adaptations to extreme environmental changes such as famine, war, and poverty that were then passed on to future generations. For instance, in utero exposure to famine during the Dutch Hunger Winter that occurred during 1944–1945 resulted in an increased risk of metabolic diseases, such as T2DM, in affected offspring, which was further passed on to future generations [101]. Researchers hypothesize that when exposed to stressful environments including famine, certain epigenetic modifications were favored to improve survival rates [102]. When these modifications are passed down to generations who are not exposed to the same environments, the once advantageous adaptations can then increase susceptibility to metabolic disease.

The consequences of previous generations are not set in stone, as another quality of epigenetics is that it is reversible. The manner in which exposure to toxins and stress can alter epigenetic modifications is the same manner in which positive factors can attenuate these modifications. Interventions of a healthy diet and exercise can go farther by rectifying epigenetic dysregulations associated with CVD [103]. However, these interventions may not be suitable nor maintainable for certain populations. This necessitates the pivoting of treatment to modern epigenetic medicine.

Though there are defined guidelines and pharmacological therapies for the treatment of CVD, several studies have shown that polypharmacy decreases patient adherence and increases drug interactions, especially in older populations [104,105,106]. There can also be adverse side effects to these medications that have harmful consequences. For example, many drugs used for cardiovascular health such as diuretics cause electrolyte imbalances, which can result in life-threatening arrhythmias if not closely monitored [107,108]. It is typical for patients to be on these traditional CVD medications long-term or even for the rest of their lives [109,110]. Therefore, it is vital to assess the pharmaceuticals’ mechanisms of action and their associated side effects to pair the patient with the most suitable therapy. A few traditional medications have also exhibited the additional capacity to modulate epigenetic factors. As these dual acting medications are rare, it is vital to seek medications that directly target epigenetic modifications. These medications, also known as epidrugs, are an emerging class of therapeutics that must be investigated for their value in the treatment of CVD. By addressing CVD at an epigenetic level, epidrugs offer promise for future therapeutics. Below is a comprehensive list of traditional medications that are currently available, examples of those with epigenetic action, and potential epigenetic medications for the treatment of patients with CVD.

## 4. Traditional CVD Medications

### 4.1. Statins

3-hydroxy-3-methylglutaryl coenzyme A (HMG-CoA) reductase inhibitors, also known as statins, are a common class of medications prescribed to patients for the prevention and treatment of ASCVD. The primary prevention method of statins is to reduce endogenous cholesterol production via inhibiting the rate-limiting step of mevalonate synthesis [111]. By inhibiting the production of this cholesterol precursor, cholesterol concentrations in the plasma will decrease. The secondary prevention method includes a reduction in inflammation, decreased activation of platelets, and stabilization of atherosclerotic plaques [112]. As there is a lowered activation of the cholesterol biosynthetic pathway due to statins, there are these beneficial pleiotropic effects. Medical practitioners refer to the 2019 ACC/AHA Guideline on the Treatment of Blood Cholesterol to Reduce ASCVD Risk for prescribing direction [113].

Although statin therapy is proven to be effective in the management of dyslipidemia and the reduction in mortality in patients with CVDs, poor adherence due to their side effects such as statin-related myopathies, potential liver damage, and memory loss have proven to be limiting factors for the use of this class of medication [114]. There is also evidence of the onset of T2DM with statin therapy; however, the findings of these studies are still controversial [75,115,116]. Specifically, characteristics such as the type of statin—for example, rosuvastatin or atorvastatin, —age, and other risk factors have been exhibited to contribute to this onset [117]. A mechanism that can contribute to both the onset of T2DM and the common myopathic side effects of statins is the inhibition of byproducts from the mevalonate pathway that play roles in mitochondrial health, calcium channel activity, and insulin signaling [118,119,120]. Together, these are significant factors that should be considered when choosing an appropriate CVD therapy for each patient.

One venue through which statin treatment can epigenetically address CVD is by promoting histone acetylation. In cancer cells, a panel of statins were tested for their ability to affect histone acetylation, primarily histone 3, compared to known HDAC inhibitors (HDACi) Trichostatin A (TSA) and valproic acid (VPA) [121]. Several statins exhibited the capacity to impede HDAC activity; however, lovastatin was found to most effectively inhibit HDACs 1, 2, and 3 via competitive inhibition. The implications of these HDACs in CVD are still under investigation; however, there are studies that suggest a cardioprotective effect of HDACis [122,123,124].

Another means by which statins have demonstrated epigenetic effects is by altering the expression of miRNAs. The miRNA miR-34a was observed to be upregulated in patients with CVD and in animal models experiencing cardiac complications [125,126,127]. Being tied to CVD, aging, and inflammation, miR-34a is a prime therapeutic target for the improvement of cardiovascular health [128,129]. A study by Tabuchi et al. revealed that atorvastatin can decrease miR-34a expression in CAD patient-derived endothelial progenitor cells [130]. Conversely, in cancer cell lines, simvastatin was described to reduce miR-34a [131]. The epigenetic effects of statins have already played a central role in recent cancer research and their promising results are of great inspiration to further expand their research to potentially combine statin and epigenetic therapy for the treatment of CVDs.

### 4.2. Calcium Channel Blockers

There are two major sub-categories of Calcium channel blockers (CCB): dihydropyridine CCBs (DHP CCB) and non-dihydropyridine CCB (Non-DHP CCB). DHP CCBs comprise pharmaceuticals such as amlodipine and nifedipine, whereas Non-DHP CCBs include verapamil and diltiazem [132]. DHP CCBs act by dilating smooth vascular tissue through the inhibition of the long-lasting, also known as L-type, voltage-dependent calcium channels [133]. Conversely, Non-DHP CCBs work at inhibiting the calcium channels in the heart, specifically at the sinoatrial and atrioventricular nodes, to reduce the heart’s contractility and rate as well as dilate blood vessels [134]. These differences in the mechanism of action between the subcategories can heavily influence practitioner prescription decisions. Typically, CCBs are used for the treatment of angina and have demonstrated benefits in morbidity and mortality when used to treat hypertension and other CVDs, such as arrhythmias [135,136]. CCBs can also be useful after myocardial infarction (MI); however, certain criteria including a prior attempt with combinatorial therapy of a beta blocker and an angiotensin-converting enzyme (ACE) inhibitor should be met before proceeding [137]. Conversely, it is not recommended for CCBs to be used in patients with heart failure (HF), as more recently, they have not demonstrated any beneficial therapeutic effects or, in more perilous cases, have caused harm [138].

Typical side effects patients experience while taking CCBs are edema, nausea, headache, and dizziness [139]. Some studies have discussed the potential association between CCBs and an increased risk of MI, gastrointestinal hemorrhage, and cancer [140,141,142]. These associations are still unclear, as other publications have described no association [143,144]. Further research on these potential associations is necessary, especially pertaining to sub-category-specific associations. Another consideration for prescribing CCBs is their contraindications and interactions. For example, certain Non-DHP CCBs are not recommended for patients with atrial fibrillation, atrioventricular blocks, or low blood pressure and can raise digoxin levels [145].

Aside from their vasodilatory capacities, some CCBs can also exert epigenetic effects. There is evidence of CCBs reducing the biological age via epigenetic clocks by maintaining the status of DNA methylation [146,147]. Modified DHP CCBs known as 1,4-dihydropyridines (1,4-DHP) have exhibited the ability to activate SIRTs [148]. A study by Manna et al. evaluated the 1,4-DHP derivative 3,5-diethoxy carbonyl-4-(4-nitrophenyl)-2,6-dimethyl-1,4-dihydropyridine (DHP-8) in activating SIRT1 via protein docking, modeling, and binding abilities [149]. DHP-8 was identified to be an allosteric enhancer of SIRT1 leading to the increased binding of SIRT1 to its targets and its cofactor NAD^+^. More recently, another 1,4-DHP, MC2789, was found to activate SIRT3 in cancer cell lines, resulting in elevated activation of manganese-dependent superoxide dismutase (MnSOD) [150]. This is especially advantageous, as MnSOD protects the mitochondria from oxidative stress and subsequent dysfunction [151]. The cardioprotective, antioxidant qualities of these Sirtuins and their link to longevity and metabolic regulations make them promising therapeutic targets in the treatment of CVD [152,153].

### 4.3. Beta Blockers

Beta blockers are a diverse collection of drugs with varying pharmacokinetic and pharmacodynamic properties. They work by primarily targeting the receptors in the heart, beta-1 (β_1_) receptors, but can also target other tissues such as smooth and skeletal muscle due to their versatile locations, beta-2 (β_2_) receptors [154]. Through the blocking of these beta receptors, there can be a slowing of the heart rate, vasodilation, and an extension of refractory times in the heart [155]. Beta-3 (β_3_) receptors were commonly targeted for their utilization with weight loss and bladder control, but recently, they have exhibited therapeutic value in the treatment of heart failure [156]. They have proven positive effects on mortality and CVD when used in people with heart failure or acute MI; however, their merit as a first-line therapeutic is under deliberation [157,158].

One study found that in the group of patients who had previously experienced an MI and those who were diagnosed only with coronary artery disease (CAD) without an MI, no significant difference was observed between the groups that received either placebo or beta blockers [159]. In a recent meta-analysis, evidence for the benefits of beta blockers was described to be heavily dependent on the context of the patient condition such as the left ventricular ejection fraction (LVEF) capacity, the diagnosis of T2DM, or the diagnosis of chronic kidney disease [160]. There are also common side effects such as hyperglycemia, diarrhea, and dizziness to consider before prescribing [161,162]. Beta blockers are also observed to modulate epigenetic regulations. Recently, a study conducted in zebrafish demonstrated the ability of the beta blocker atenolol to reduce the methylation of DNA [163]. This was exhibited to be mediated by atenolol’s binding and inhibition of DNMT1. The connection between the regulation of DNA methylation and CVD is well described; however, specifics on the therapeutic targeting of DMNTs still remain elusive [164,165]. Other epigenetic modulators that the beta blockers metoprolol and atenolol were described to impact were the expressions of miR-19a, -101, and let-7e from responsive and nonresponsive patients [166]. By revealing which miRNAs are correlated with a response to beta blockers, there can be the discovery of new drug epigenetic mechanisms, potential therapeutic targets, and methods for guiding the prescription of beta blockers in a patient-specific manner. Further investigation into the potential epigenetic mechanisms of beta blockers and their promise in precision medicine is urged.

### 4.4. ACEIs/ARBs

Angiotensin I enzyme inhibitors (ACEIs) and angiotensin II receptor blockers (ARB) are recognized as first-line hypertension therapeutics for patients who are between the ages of 18 and 60, are diabetic, have a risk or diagnosis of chronic kidney disease, or are not black/African American [167]. ACEIs and ARBs both block different parts of the renin-angiotensin-aldosterone system (RAAS) to reduce blood pressure. ACEIs were found to reduce MI, CVD-related, and all-cause mortality in patients with heart failure, whereas ARBs were more advantageous in the context of stroke and end-stage renal disease [168,169]. More recently, a meta-analysis conducted by Awad et al. described how ACEIs facilitated the reduction in the expression of inflammatory markers such as tumor necrosis factor α (TNF-α) and C-reactive protein (CRP), while ARBs only reduced interleukin 6 (IL-6) expression [170]. The differences in the mechanisms of action between these classes can be what are responsible for both the deviation in efficacy and for the emergence of side effects.

ACEIs block the Angiotensin I enzyme to inhibit the conversion of Angiotensin I to Angiotensin II, which leads to vasoconstriction and a decrease in sodium and water reabsorption [171]. Since ACE is produced in the lungs, the inhibition of this enzyme prevents the breakdown of an inflammatory mediator, bradykinin. The increased buildup of this mediator leads to a dry, hacking cough and, in severe cases, angioedema [172]. While the side effects of coughing may not seem perilous, this can affect quality of life and cause patients to discontinue this medication. Most patients describe the cessation of the cough after a couple months of treatment; however, if persistent, other therapeutic options such as ARBs should be considered [173]. Instead of inhibiting ACE, ARBs inhibit the binding of angiotensin II by acting upon the receptors, predominantly Angiotensin II type 1 (AT_1_) receptors, to reduce blood pressure [174,175]. Patients on this class of drugs do not experience a dry cough, and other reported side effects are milder, making it more tolerable than ACEIs [176]. As both classes offer benefits against CVD, their side effects may be the determining factor for prescription decisions.

In addition to their attenuation of hypertension, ACEIs and ARBs can also exhibit epigenetic action. The ARB losartan was found to alter histone modifications in diabetic mice when observing its benefits in diabetic nephropathy [177]. Both HATs and HDACs were found to have modulated gene expression under losartan treatment, which was associated with decreased inflammatory markers and improved kidney function. As the kidney is a central component of the RAAS, recovering its function and mitigating its inflammation are critical in ameliorating hypertension and subsequently CVD. Another study demonstrated that losartan treatment attenuated the adipose-specific hypomethylation of AT_1_ receptor genes and the upregulation of receptor expression in high-fat-diet rats [178]. Losartan treatment not only reduced the blood pressure of the high-fat-diet-fed rats that persisted even after 16 weeks of the discontinuation of the treatment but also improved the metabolic lipid profiles and inhibited weight gain. This exemplifies that by addressing epigenetic dysregulations, the prescription of medications does not have to be perpetual.

## 5. Epigenetic CVD Medications

### 5.1. HDAC Inhibitors (HDACIs)

Histone deacetylase (HDAC) is an enzyme that removes acetyl groups from the lysine residues of both the histone and nonhistone proteins. HDACs are divided into zinc (Zn^2+^)-dependent and nicotinamide adenine dinucleotide (NAD^+^)-dependent mechanisms of deacetylation [179]. Zn^2+^-dependent HDACs encompass Class I, Class II, and Class IV. Class III HDACs are NAD^+^-dependent and known as Sirtuins (SIRTs), which will be elaborated on later. When histones are deacetylated, usually at lysine and arginine residues, they hold an overall positive charge, leading to tighter interactions with the negatively charged DNA [180]. As a result, chromatin condensation occurs, restricting transcription factors and other transcription machinery from accessing the DNA. In addition to transcriptional changes at the site of deacetylation, HDACs can interact with other epigenetic mediators in complexes or independently to direct further chromatin remodeling [181].

HDACs play vital roles in several physiological functions not limited to cell proliferation and survival, insulin resistance, gluconeogenesis, as well as cardiac myocyte and endothelial cell growth and function [124,182]. HDACs are observed to contribute to the development and progression of atherosclerosis [183]. They are also exhibited to regulate the signaling cascades in HF [184]. HDACs are crucial in the expression of leptin, a hormone that promotes satiety and can contribute to obesity, which is a major risk factor in the development of CVDs [185,186]. The inhibition of HDACs in diabetic and high-fat-diet-fed mice has led to improved cardiac function, reduced oxidative inflammation, decreased weight, and decreased serum glucose [187,188]. A summary of HDACIs, STACs, and HATIs and traditional drugs with comparable mechanisms of action is depicted in Table 1. With such a myriad of functions, HDACs and their inhibition open a huge venue for exploring various treatment options for CVDs.

#### 5.1.1. Valproic Acid

Known for its original indication as an anticonvulsant, valproic acid is a staple HDACI [189,190]. Valproic acid is an inhibitor of HDACs 1, 2, 3, and 8 from class I and HDACs 4, 5, 7, and 9 from class II, with a preference for class I [191]. By inhibiting HDACs, valproic acid can offer new options for CVD treatment. A study was conducted with valproic acid to determine if the prevention of hypertension was possible by the acetylation of the mineralocorticoid receptor (MR) in hypertensive rats [192]. It was identified that treatment with valproic acid inhibited HDAC3, resulting in the increased acetylation of MR and subsequent decreased transcriptional activity. MR controls the salt balance and water homeostasis by regulating aldosterone, whose primary function is to increase sodium and water retention [193]. A high retention of sodium and water increases intravascular volume and pressure, contributing to hypertension and fluid retention in heart failure patients. In another study, valproic acid was investigated for its potential to attenuate the remodeling of the heart in a mouse model where an isoform of cAMP-responsive element modulator (CREM) associated with atrial fibrillation was induced [194]. Valproic acid demonstrated benefits for atrial remodeling through a reduction in fibrosis, thrombosis, and delaying the onset of atrial fibrillation. This can offer significant promise in CVD treatment, as atrial fibrillation is known to increase CVD and death [195]. Valproic acid has also exhibited direct protective effects in cardiomyocytes after MI [196]. Recently, the same lab conducted a meta-analysis on two retrospective studies under a similar circumstance after MI to evaluate the risk of HF between patients that had active prescriptions for valproic acid when MI occurred and those who did not [197]. This meta-analysis provided some insight into how those who had been prescribed valproic acid, specifically at a higher dosage, possibly experienced a protective effect against HF. While this can indicate the potential of valproic acid in HF, further clinical studies are warranted to fully elucidate the effects. Cumulatively, this preliminary evidence illustrates the merit of the consideration of valproic acid as a treatment for CVD.

#### 5.1.2. Sodium Phenylbutyrate

Sodium phenylbutyrate, a 4-phenyl butyric acid (PBA), is a pan-HDACI that also serves as a chemical chaperone in facilitating the reduction in ROS [198]. An earlier account of PBA’s potential in CVD treatment arose when it was implemented in a study to observe if it can mitigate the toxic effects that the anticancer drug Adriamycin induced in the hearts of mice [199]. Impressively, PBA was able to reduce several cardiac insults elicited by Adriamycin treatment such as an improved ejection fraction, reduced cardiac structural defects, and increased MnSOD activity. Another study demonstrated that through its chaperone function, PBA facilitated the decrease in the unfolded protein response, which is a detriment of myocardial reperfusion therapy after MI [200]. This study exhibited that by inhibiting endoplasmic reticulum (ER) stress, PBA was able to improve cardiac health. Through a similar mechanism, Wu et al. revealed that PBA treatment reduced ER stress and ameliorated the remodeling of the pulmonary arteries in hypertensive rats [201]. Conversely, there are also studies that describe PBA as exacerbating cardiac dysfunction and hypertrophy, especially when compared to other epidrugs [202]. The use of PBA in CVD treatment still requires further inquiry; however, these initial findings have provided a positive basis for future opportunities.

#### 5.1.3. Vorinostat

Suberoylanilide hydroxamic acid (SAHA, also known as Vorinostat) is an HDACI that is part of the hydroxymate group that also encompasses other HDACIs such as Givinostat, Panobinostat, and Trichostatin A [203]. It works primarily by inhibiting Class I and Class II HDACs and by competitively binding to the catalytic site. Vorinostat’s anti-inflammatory properties and inhibitory effect on apoptosis can be of potential use for CVD treatment, especially when HDACIs have been shown to prevent cardiac remodeling [204]. In rabbits with ischemic reperfusion injury and aged mice with diastolic failure, vorinostat attenuated and even reversed cardiac dysfunction [205]. Another study demonstrated that by vorinostat’s inhibition of HDAC6, there was a boosted cardiac pump function and increased heat shock protein expression in MI rat models [206]. Heat shock proteins are responsible for protein maintenance and have arisen as a potential target for the treatment of CVD [207]. Vorinostat has also exhibited promise in the treatment of hypertension. In samples from patients with pulmonary arterial hypertension (PAH), HDAC1 and HDAC8 were observed to be increased [208]. To investigate potential treatment options, pulmonary artery-isolated adventitial fibroblasts were made hypoxic to mimic PAH as well as treated with vorinostat and were found to markedly lower systolic pressure, mean pulmonary arterial pressure, and cardiac hypertrophy. Together, these findings present the prospective efficacy of vorinostat in CVD therapeutics.

#### 5.1.4. Trichostatin A

Similar to Vorinostat, Trichostatin A (TSA) is a pan-HDAC inhibitor that works both epigenetically and non-epigenetically by inhibiting mitogen-activated protein kinase (MAPK) signaling [209,210]. Notably, the activation of MAPK signaling pathways has been thoroughly implicated with cardiac hypertrophy, remodeling, and even the development of atherosclerosis [211,212]. This can potentiate two approaches by which TSA can be utilized for CVD intervention. Yang et al. put these approaches to practice as they elucidated the MAPK regulation of nitric oxide (NO) production in the context of ischemia and reperfusion [213]. They discovered that HDAC1 mediated the reduced acetylation state of the gene promoter for endothelial nitric oxide synthase (eNOS) and that treatment with TSA rescued NO production. In CVD, eNOS and NO play pivotal roles in blood pressure regulation, making them attractive therapeutic targets [214].

Other downstream targets of the MAPK signaling cascade include matrix metalloproteinases (MMP) that are responsible for the degradation of the extracellular matrix and subsequent fibrosis during cardiac remodeling [215]. Further, inflammation can exacerbate fibrosis, as inflammatory markers such as Interleukin-1 (IL-1), Interleukin-6 (IL-6), and nuclear factor-κB (NF-κB) can upregulate MMP expression. This sets the stage for an investigation into the mechanisms regulating cardiac fibrosis. In a study using mouse cardiac fibroblasts, it was revealed that Angiotensin II activated HDAC1 and HDAC2, resulting in the initiation of fibrosis [216]. Treatment with TSA proved to be advantageous against fibrosis by inhibiting the binding of NF-κB to the promoter region of MMP9 and Interleukin-18 that induces their expression. By addressing inflammation, MAPK pathway activation, and epigenetic modifications, TSA sets a foundation for continued research into the benefits of epidrugs in the treatment of CVD.

### 5.2. SIRT Family + Sirtuin-Activating Compounds (STACs)

Originally identified in yeast, SIRTs are Class III histone deacetylases that are nicotinamide adenine dinucleotide NAD^+^-dependent. In humans and other mammals, there are 7 SIRTs, each consisting of different subcellular localization, enzymatic activity, and binding targets [217]. The SIRT family members are divided into four classes: Class I (consisting of SIRT1, SIRT2, and SIRT3), Class II (SIRT4), Class III (SIRT5), and Class IV (SIRT6 and SIRT7) [218]. Specifically, SIRTs 1, 2, 3, 6, and 7 have been identified to contribute to cardiac regulations [219]. As Sirtuins are implicated across aging, metabolic diseases, and CVD, the utilization of compounds that can modulate their activity can offer alternative epigenetic therapeutic options [220].

#### Resveratrol

Resveratrol is a naturally occurring polyphenol that was originally discovered in the roots belonging to *Veratrum grandiflorum* and *Polygonum cuspidatum*, plants that were used in folk medicine [221]. Presently, resveratrol is found to be abundant in a multitude of fruits and legumes. Resveratrol is a well-established epidrug, primarily for its prowess in metabolic disease. As CVD is a metabolic disease and other metabolic diseases such as obesity and T2DM are significant risk factors for CVD, the potential use of resveratrol for CVD treatment is well founded. In a clinical study evaluating the effect of resveratrol on platelet aggregation in participants with T2DM, it was revealed that resveratrol treatment decreased platelet adhesion, aggregation, and thromboxane A_2_ production in both the healthy and diabetic participants [222]. Other benefits from resveratrol treatment included an improved expression of glucose metabolism enzymes and inhibited thrombus formation, all while not impacting the viability of the platelets. One limitation of prolonged antiplatelet therapy for the prevention of CVD is the risk of increased bleeding, especially in older patients; resveratrol may provide a more viable alternative.

Recently, a clinical study over six months investigated the therapeutic value of resveratrol alongside traditional medications in hypertensive participants [223]. The effects of combinatorial therapy with resveratrol on cardiac remodeling were modest; however, in the serum, there were increased levels of SIRT3, superoxide dismutase (SOD), and klotho proteins. This can be a strong indicator of the inhibition of oxidative stress, the enhancement of mitochondrial function, and the attenuation of mineral reabsorption and excretion, which can potentially continue even after halting resveratrol treatment [224,225,226]. Additionally, the axis between resveratrol, SIRT3, and SOD is further demonstrated in a study using the cell lines of patients with mitochondrial complex I deficiency [227]. It was elucidated that resveratrol treatment increased SIRT3 expression and activity, leading to an increase in SOD2 expression and activity, and ultimately mediated a reduction in oxidative stress.

Another Sirtuin that has presented promise in the treatment of CVD is SIRT1. Its expression and activation have been linked to decreasing inflammation, modulating NO production, and improving metabolic parameters [228]. In rats modeling coronary microembolization (CME), resveratrol was assessed for the potential protective roles it may play in cardiac health [229]. Resveratrol treatment demonstrated an amelioration of cardiac function in CME rats via increased SIRT1 expression that directly led to reduced cardiomyocyte apoptosis. A similar effect of resveratrol’s ability to reverse cardiac dysfunction was exhibited in cardiomyocytes of diabetic cardiomyopathic mice [230]. This reversal included improving the ejection fraction, the heart’s blood volume capacity, and reducing cardiac fibrosis, which was identified to be mediated by SIRT1. Overall, these mechanisms of resveratrol accentuate the advantages of resveratrol in the treatment of CVD.

### 5.3. HAT Inhibitors (HATIs)

As opposed to HDAC inhibitors, HAT inhibitors regulate acetylation by inhibiting the enzymes that add the acetyl groups. The acetylation of histone tails neutralizes the positively charged residues to impede the tight interaction between histone proteins and DNA, thus promoting gene transcription. HATs are divided into two major classes: Type A, the nuclear HATs and Type B, the cytoplasmic HATs [231]. Similar to HDACs, HATs can also interact with other epigenetic modulators as well as participate in complexes [232]. HATs are also described to contribute to the development and progression of CVD [233,234,235]. As we have discussed the role of HDACs in CVD therapeutics, we must also look at the other side of the coin.

#### Curcumin

Curcumin is a polyphenolic compound found in turmeric, a common spice historically used in Southeast Asian cultures, whose pharmacological effects have been studied in several diseases like cancer, arthritis, and other inflammatory diseases [236]. Curcumin’s primary HAT target is p300 [237,238]. However, curcumin has demonstrated more versatile epigenetic capacities such as inhibiting HDACs, modulating DNA methylation, and altering miRNA expression [239]. Curcumin’s capacity to reduce inflammation, modulate signaling cascades, and work at an epigenetic level makes it a viable therapeutic option for CVD treatment.

Using a hypertensive rat model and an MI rat model, Morimoto et al. ventured to observe the potential effects of curcumin on these mediators of heart failure [240]. In both rat models, curcumin treatment demonstrated an enhancement of ventricular function and mitigated cardiomyocyte hypertrophy. These benefits were proposed to be mediated by curcumin’s inhibitory effect on p300, resulting in the decreased acetylation and activation of the cardiac transcription factor GATA4 in the hypertensive rat model. This mechanism was expanded upon in a more recent study where the same group sought to investigate curcumin’s effects in left ventricular hypertrophy induced by hypertension [241]. Again, curcumin exhibited cardioprotective effects by reducing fibrosis, hypertrophy response gene expression, and the cell diameter of cardiomyocytes. This was accompanied by reduced GATA4 acetylation due to p300 inhibition. Activated GATA4 is known to associate with other transcription factors such as serum response factor (SRF) and NK2 homeobox 5 (NKX2-5) to initiate the pathway for cardiac hypertrophy-related gene expression [242]. Curcumin has also exhibited antifibrotic potential in the heart of MI mouse models; to further understand the mechanism, LPS-stimulated Raw 264.7 cells treated with curcumin demonstrated an inhibition of inflammation through the downregulation of interleukin-1β (IL-1β), interleukin-18 (IL-18), IL-6, and TNF-α [243]. Specifically, the decrease in IL-18 was identified to inhibit transforming growth factor-β1-phosphorylated-small mothers against decapentaplegic 2/3 (TGFβ1-*p*-SMAD2/3) signaling, responsible for cardiac fibrosis, downstream [244]. Curcumin can also be beneficial in CVD intervention by ameliorating risk factors. In a meta-analysis investigating the lipid-lowering potential of turmeric and its star compound curcumin, TG and LDL cholesterol were significantly reduced in patients that received either treatment [245]. Additionally, no severe adverse effects were reported. With the potential to attenuate cardiac fibrosis and hypertrophy and reduce inflammation, curcumin adds to the list of epidrugs that can be advantageous in the treatment of CVD.

### 5.4. Interventional Clinical Trial Updates of Epigenetic Drugs

A list of interventional clinical trials of epigenetic drugs in the treatment of CVDs is compiled in Table 2. Even though many clinical studies are completed, the publication of those studies’ outcomes is still required. One clinical trial (NCT03903302; https://clinicaltrials.gov/ (accessed on 7 November 2023)) investigated the pharmacokinetics of valproic acid in obese, borderline hypertensive but otherwise healthy subjects. Another clinical trial (NCT00774306; https://clinicaltrials.gov/ (accessed on 7 November 2023)) evaluated the effects of valproic acid for its use as an antiepileptic medication in patients with subarachnoid hemorrhages. Serum cholesterol, non-HDL cholesterol, HDL cholesterol, lipoprotein A, and C-reactive protein were measured at baseline and post-treatment with valproic acid or another antiepileptic medication. This study was terminated due to inadequate follow-up, and no further analysis was conducted on this data. Other studies investigating valproic acid have been withdrawn due to sponsorship termination, are of unknown status, possibly due to recruitment, or are ongoing. Interestingly, there are no recorded studies of sodium phenylbutyrate, vorinostat, or Trichostatin A in the context of CVD. This can present a lucrative opening for the further research of HDACis as potential interventions for CVD.

There have been clinical trials within the STAC class focusing mainly on resveratrol. A clinical study (NCT01449110, https://clinicaltrials.gov/ (accessed on 7 November 2023)) explored the safety and efficacy of resveratrol-enriched grape extract in patients for primary and secondary cardiovascular prevention. This clinical trial provided a basis for intervention with CVD at two time points of 6 months and 1 year. At the 6-month time point, grape extract enriched with resveratrol significantly reduced ApoB, LDL, and oxidized LDL levels in the serum samples compared to placebo [246]. The enrichment of grape extract with resveratrol was key, as grape extract alone did not present with as impressive effects. After a year, grape extract enriched with resveratrol was found to decrease IL-1β, TNF-α, C–C motif chemokine ligand 3 (CCL3), and IL-6 in the peripheral blood mononuclear cells of T2DM patients with hypertension [247]. Conversely, in stabilized patients with CAD, grape extract enriched with resveratrol reduced plasminogen activator inhibitor type 1, which is conducive to blood clotting, while increasing the anti-inflammatory cytokine adiponectin in serum [248]. Most significantly, there were no observed adverse events across the clinical study. In another trial inspired by the “French Paradox” of high saturated fat diets and low CVD risk, the Effects of Resveratrol Supplements on Vascular Health in Postmenopausal Women (NCT01564381, https://clinicaltrials.gov/ (accessed on 7 November 2023)) sought to seek if a novel form of resveratrol can provide cardiovascular benefits. Different formulations of Resveratrol have also been explored, but so far, no data have been reported from any of these trials.

A clinical study (NCT01225094, https://clinicaltrials.gov/ (accessed on 7 November 2023)) investigated the potential of an acute prophylactic treatment of 4 g of curcumin daily in patients that underwent elective abdominal aortic aneurysm repair to prevent associated complications [249]. It was revealed that curcumin supplementation unexpectedly increased the risk of acute kidney injury compared to placebo or had no effect on targeted biomarkers such as c-reactive protein, interleukin-18, and creatine. Possible reasons for these outcomes that are contrary to a previous study are the formulation of the curcumin supplement, the duration of the intervention period, and the concentration of each dose provided [250]. Other completed clinical trials with curcumin have yet to be published, and further studies are warranted to elucidate its therapeutic potential. There is an anticipation for new clinical studies being implemented to delve deeper into the prowess that these epidrugs have against CVDs.

## 6. Conclusions

As the demand for individualized and optimized therapy continues to increase, epigenetic modifications can help bridge the gap. Currently, novel epigenetic regulations are being discovered in their contributions to the development and progression of several diseases including obesity, metabolic dysfunction-associated steatotic liver disease, osteoporosis, and, of course, CVD [251,252]. This begins to illustrate the major impact that epigenetics can have on our current medical care practices in CVDs. As CVD maintains its status as the leading cause of death globally, it is evident that traditional CVD therapeutics alone are not enough to overcome this villain. In the battle against cancer, the combinatorial therapeutics of chemotherapy medications and epidrugs are ongoing and encourage this two-pronged approach [253]. This approach can also be applied in the CVD scenario, although contraindications and drug interactions must be thoroughly evaluated for their safety prior to prescription. The deficit of knowledge of the potential synergistic effects of traditional CVD medications and epidrugs provides an exciting opportunity to not only advance the field of medicine, but to also find new ways to improve patients’ quality of life.

## Figures and Tables

**Table 1:** Summary of epigenetic and traditional drugs with similar mechanisms of action.

Epigenetic Drugs	Mechanism of Action	Similar Traditional Drug
Valproic Acid	Decreases the transcription of the mineralocorticoid receptor to reduce sodium and water retention, inhibits cardiac remodeling, and attenuates atrial fibrillation	Statins, ARBs, ACEIs, Beta Blockers
Sodium Phenylbutyrate	Increases MnSOD activity, mitigates reperfusion therapy post-MI, and reduces ER stress	Calcium Channel Blockers
Vorinostat	Anti-inflammatory, prevents cardiac remodeling, attenuates cardiac dysfunction, lowers systolic and pulmonary arterial pressure, inhibits cardiac hypertrophy	ACEIs, Statins, ARBs
Trichostatin A	Decreases cardiac hypertrophy, inhibits MAPK pathway activation, decreases inflammatory marker expression, and improves eNOS expression	Statins, ARBs
Resveratrol	Anti-inflammatory, inhibits cardiomyocyte apoptosis, inhibits platelet adhesion and aggregation, increases SOD expression, inhibits oxidative stress, and improves mitochondrial function	Statins, Calcium Channel Blockers, Beta Blockers
Curcumin (Turmeric)	Inhibits miRNA expression, mitigates cardiac hypertrophy, inhibits IL-6, IL-1β, IL-18, and TNF-α, reduces serum lipid levels, and reduces CVD risk factors	Statins, ACEIs, ARBs,

**Table 2:** Interventional Clinical Trials of Epigenetic Drugs in the Treatment of CVD.

Drug	Condition/Disease	Status	NCT Number	Phase
**HDACi**				
Valproic Acid	Thrombosis	Completed	NCT03903302	Phase 1
	Subarachnoid Hemorrhage	Terminated	NCT00774306	N/A
	Anoxic Encephalopathy; Cardiac Arrest; Status Epilepticus;	Completed	NCT02056236	N/A
	Brain Injuries, Acute; Brain Injuries, Traumatic; Brain Ischemia; Brain Hypoxia; Hypoxia-Ischemia, Brain; Heart Arrest; Stroke; Intracranial Hemorrhages; Coma; Persistent Vegetative State;	Not Yet Recruiting	NCT06081283	Phase 4
	Acute Ischemic Stroke	Recruiting	NCT06020898	Phase 2
	Cardiac Valve disease; Coronary Brain Artery Disease; Organ Failure, Multiple;	Unknown Status	NCT03825250	Phase 1, Phase 2
	Cerebral Aneurysm	Unknown Status	NCT01460563	N/A
	Acute Kidney Injury; Ischemia Reperfusion Injury	Withdrawn	NCT04531579	Phase 2
	Acute Kidney Injury; Ischemia Reperfusion Injury	Withdrawn	NCT04531592	Phase 2
	Post Cerebral Hemorrhage	Completed	NCT01115959	Phase 4
	Cardiac Arrest	Unknown Status	NCT01083784	Phase 4
	Cardiac Arrest; Status Epilepticus	Recruiting	NCT05756621	
	Pulmonary Arterial Hypertension	Recruiting	NCT05224531	Phase 2
Sodium Phenylbutyrate	No Recorded Studies			
Vorinostat	No Recorded Studies			
Trichostatin A	No Recorded Studies			
**STAC**				
Resveratrol	Cardiovascular Diseases	Completed	NCT01449110	Phase 2
	Cardiovascular Disease	Completed	NCT01564381	Phase 1, Phase 2
	Hypercholesterolemia	Completed	NCT02409537	Phase 2
	Vascular System Injuries, Lipid Metabolism Disorders, Endothelial Dysfunction;	Completed	NCT01668836	N/A
	Peripheral Artery Disease	Completed	NCT02246660	N/A
	Metabolic Syndrome, Coronary Artery Disease	Completed	NCT02137421	N/A
	Cardiovascular Diseases; Autonomic Nervous System; Polyphenols;	Recruiting	NCT06020313	N/A
	Coronary Artery Disease; Diabetes Mellitus, Type 2;	Active, Not Recruiting	NCT03762096	Phase 1, Phase 2
	Pulmonary Disease, Chronic Obstructive;	Completed	NCT02245932	N/A
	Chronic Obstructive Pulmonary Disease	Completed	NCT03819517	N/A
	Peripheral Artery Disease	Completed	NCT03743636	Phase 3
	Hypertension; Vascular Resistance;	Terminated	NCT01842399	Phase 1, Phase 2
	Coronary Artery Disease; Endothelial Dysfunction; Menopause	Recruiting	NCT05808387	N/A
	Cardiovascular Disease; Atherosclerosis; Inflammation;	Unknown Status	NCT02998918	Phase 2
	Autonomic Nervous System Disease; Blood Pressure; Coronary Artery Disease; Heart Rate;	Completed	NCT06095635	N/A
	Congestive Heart Failure Chronic	Completed	NCT03525379	Phase 2
	Dilated Cardiomyopathy	Unknown Status	NCT01914081	Phase 3
	Coronary Artery Disease; Metabolic Syndrome	Completed	NCT02137421	N/A
	Coronary Artery Restenosis; In-stent Coronary Artery Restenosis; In-stent Restenosis;	Withdrawn	NCT05093244	N/A
	Diastolic Heart Failure; Heart Failure with Preserved Ejection Fraction; Hypertension; Hypertensive Heart Disease; Oxidative Stress;	Completed	NCT01185067	Phase 1
	Hypertension in Pregnancy; Intrauterine Growth Restriction; Pre-Eclampsia; Pre-Term;	Completed	NCT04633551	N/A
**HATi**				
Curcumin	Cardiovascular Disease	Completed	NCT02088307	N/A
	Abdominal Aortic Aneurysm; Acute Kidney Injury;	Completed	NCT01225094	Phase 2, Phase 3
	Coronary Artery Disease; Inflammation; Oxidative Stress;	Unknown Status	NCT04458116	N/A
	Cardiovascular Disease; Atherosclerosis; Inflammation;	Unknown Status	NCT02998918	Phase 2
	Cardiovascular Abnormalities; Type 2 Diabetes Mellitus;	Unknown Status	NCT01052597	Phase 4
	Diabetes Mellitus, Type 2; Dyslipidemias; Hypertension	Recruiting	NCT05753436	Phase 2
	Cardiovascular Risk; Insulin Resistance; Pre-diabetes; Type 2 Diabetes;	Unknown Status	NCT01052025	Phase 4
	Heart Diseases; High Blood Pressure; High Cholesterol; Obesity; Type2 Diabetes;	Completed	NCT03542240	N/A
	Blood Pressure; Chronic Kidney Diseases; Hyperemia; Vasoconstriction;	Withdrawn	NCT04132648	Phase 2
	Hematoma, Subdural, Chronic;	Withdrawn	NCT03845322	Early Phase 1
	Hypertension in Pregnancy; Intrauterine Growth Restriction; Pre-Eclampsia; Pre-Term;	Completed	NCT04633551	N/A

The list is compiled from ClinicalTrials.gov using the keywords ‘cardiovascular disease’ as the condition/disease and ‘drug’, which are substituted with different epigenetic drugs as other terms. These trials are updated as of 7 November 2023. (Abbreviations: NCT indicates ClinicalTrials.gov Identifier Number; N/A indicates not available).

## Data Availability

No new data were created or analyzed in this study. Data sharing is not applicable to this article.

## References

[1] Vaduganathan M., Mensah George A., Turco Justine V., Fuster V., Roth Gregory A. (2022). The Global Burden of Cardiovascular Diseases and Risk. J. Am. Coll. Cardiol..

[2] Centers for Disease Control and Prevention Heart Disease Facts. https://www.cdc.gov/heartdisease/facts.htm.

[3] American Heart Association Statistics, Committee What Is Cardiovascular Disease. https://www.heart.org/en/health-topics/consumer-healthcare/what-is-cardiovascular-disease.

[4] Roth G.A., Mensah G.A., Johnson C.O., Addolorato G., Ammirati E., Baddour L.M., Barengo N.C., Beaton A.Z., Benjamin E.J., Benziger C.P. (2020). Global Burden of Cardiovascular Diseases and Risk Factors, 1990–2019: Update From the GBD 2019 Study. J. Am. Coll. Cardiol..

[5] Tsao C.W., Aday A.W., Almarzooq Z.I., Anderson C.A.M., Arora P., Avery C.L., Baker-Smith C.M., Beaton A.Z., Boehme A.K., Buxton A.E. (2023). Heart Disease and Stroke Statistics—2023 Update: A Report From the American Heart Association. Circulation.

[6] Powell-Wiley T.M., Poirier P., Burke L.E., Després J.-P., Gordon-Larsen P., Lavie C.J., Lear S.A., Ndumele C.E., Neeland I.J., Sanders P. (2021). Obesity and Cardiovascular Disease: A Scientific Statement From the American Heart Association. Circulation.

[7] Joseph J.J., Deedwania P., Acharya T., Aguilar D., Bhatt D.L., Chyun D.A., Di Palo K.E., Golden S.H., Sperling L.S., on behalf of the American Heart Association Diabetes Committee of the Council on Lifestyle and Cardiometabolic Health (2022). Comprehensive Management of Cardiovascular Risk Factors for Adults With Type 2 Diabetes: A Scientific Statement From the American Heart Association. Circulation.

[8] Ng A.C.T., Delgado V., Borlaug B.A., Bax J.J. (2021). Diabesity: The combined burden of obesity and diabetes on heart disease and the role of imaging. Nat. Rev. Cardiol..

[9] Amini M., Zayeri F., Salehi M. (2021). Trend analysis of cardiovascular disease mortality, incidence, and mortality-to-incidence ratio: Results from global burden of disease study 2017. BMC Public Health.

[10] Sidney S., Quesenberry C.P., Jaffe M.G., Sorel M., Nguyen-Huynh M.N., Kushi L.H., Go A.S., Rana J.S. (2016). Recent Trends in Cardiovascular Mortality in the United States and Public Health Goals. JAMA Cardiol..

[11] Lloyd-Jones D.M., Allen N.B., Anderson C.A.M., Black T., Brewer L.C., Foraker R.E., Grandner M.A., Lavretsky H., Perak A.M., Sharma G. (2022). Life’s Essential 8: Updating and Enhancing the American Heart Association’s Construct of Cardiovascular Health: A Presidential Advisory From the American Heart Association. Circulation.

[12] Diaz C.L., Shah N.S., Lloyd-Jones D.M., Khan S.S. (2021). State of the Nation’s Cardiovascular Health and Targeting Health Equity in the United States: A Narrative Review. JAMA Cardiol..

[13] Javed Z., Haisum Maqsood M., Yahya T., Amin Z., Acquah I., Valero-Elizondo J., Andrieni J., Dubey P., Jackson R.K., Daffin M.A. (2022). Race, Racism, and Cardiovascular Health: Applying a Social Determinants of Health Framework to Racial/Ethnic Disparities in Cardiovascular Disease. Circ. Cardiovasc. Qual. Outcomes.

[14] Ordovas J.M., Smith C.E. (2010). Epigenetics and cardiovascular disease. Nat. Rev. Cardiol..

[15] van Dijk S.J., Tellam R.L., Morrison J.L., Muhlhausler B.S., Molloy P.L. (2015). Recent developments on the role of epigenetics in obesity and metabolic disease. Clin. Epigenet..

[16] Kazmi N., Elliott H.R., Burrows K., Tillin T., Hughes A.D., Chaturvedi N., Gaunt T.R., Relton C.L. (2020). Associations between high blood pressure and DNA methylation. PLoS ONE.

[17] Kou M., Li X., Shao X., Grundberg E., Wang X., Ma H., Heianza Y., Martinez J.A., Bray G.A., Sacks F.M. (2023). DNA Methylation of Birthweight–Blood Pressure Genes and Changes of Blood Pressure in Response to Weight-Loss Diets in the POUNDS Lost Trial. Hypertension.

[18] Lehmann L.H., Worst B.C., Stanmore D.A., Backs J. (2014). Histone deacetylase signaling in cardioprotection. Cell. Mol. Life Sci..

[19] Finkelstein E.A., Khavjou O.A., Thompson H., Trogdon J.G., Pan L., Sherry B., Dietz W. (2012). Obesity and severe obesity forecasts through 2030. Am. J. Prev. Med..

[20] Ward Z.J., Bleich S.N., Cradock A.L., Barrett J.L., Giles C.M., Flax C., Long M.W., Gortmaker S.L. (2019). Projected U.S. State-Level Prevalence of Adult Obesity and Severe Obesity. N. Engl. J. Med..

[21] Fryar C.D., Carroll M.D., Afful J. Prevalence of Overweight, Obesity, and Severe Obesity among Adults Aged 20 and Over: United States, 1960–1962 through 2017–2018. https://www.cdc.gov/nchs/data/hestat/obesity_adult_15_16/obesity_adult_15_16.htm.

[22] Fuster J.J., Ouchi N., Gokce N., Walsh K. (2016). Obesity-Induced Changes in Adipose Tissue Microenvironment and Their Impact on Cardiovascular Disease. Circ. Res..

[23] Koenen M., Hill M.A., Cohen P., Sowers J.R. (2021). Obesity, Adipose Tissue and Vascular Dysfunction. Circ. Res..

[24] Centers for Disease Control and Prevention Strategies to Prevent Obesity. https://www.cdc.gov/obesity/strategies/index.html.

[25] Sadashiv, Modi A., Khokhar M., Sharma P., Joshi R., Mishra S.S., Bharshankar R.N., Tiwari S., Singh P.K., Bhosale V.V. (2021). Leptin DNA Methylation and Its Association with Metabolic Risk Factors in a Northwest Indian Obese Population. J. Obes. Metab. Syndr..

[26] Liu H., Du T., Li C., Yang G. (2021). STAT3 phosphorylation in central leptin resistance. Nutr. Metab..

[27] Mikula M., Majewska A., Ledwon J.K., Dzwonek A., Ostrowski J. (2014). Obesity increases histone H3 lysine 9 and 18 acetylation at Tnfa and Ccl2 genes in mouse liver. Int. J. Mol. Med..

[28] Castillo E.C., Morales J.A., Chapoy-Villanueva H., Silva-Platas C., Treviño-Saldaña N., Guerrero-Beltrán C.E., Bernal-Ramírez J., Torres-Quintanilla A., García N., Youker K. (2019). Mitochondrial Hyperacetylation in the Failing Hearts of Obese Patients Mediated Partly by a Reduction in SIRT3: The Involvement of the Mitochondrial Permeability Transition Pore. Cell. Physiol. Biochem..

[29] Romanick S.S., Ulrich C., Schlauch K., Hostler A., Payne J., Woolsey R., Quilici D., Feng Y., Ferguson B.S. (2018). Obesity-mediated regulation of cardiac protein acetylation: Parallel analysis of total and acetylated proteins via TMT-tagged mass spectrometry. Biosci. Rep..

[30] International Diabete Federation (2021). IDF Diabetes Atlas.

[31] Association A.D. (2018). Economic Costs of Diabetes in the U.S. in 2017. Diabetes Care.

[32] Dal Canto E., Ceriello A., Rydén L., Ferrini M., Hansen T.B., Schnell O., Standl E., Beulens J.W.J. (2019). Diabetes as a cardiovascular risk factor: An overview of global trends of macro and micro vascular complications. Eur. J. Prev. Cardiol..

[33] Nahmias A., Stahel P., Xiao C., Lewis G.F. (2020). Glycemia and Atherosclerotic Cardiovascular Disease: Exploring the Gap Between Risk Marker and Risk Factor. Front. Cardiovasc. Med..

[34] Bunyavanich S., Do A., Vicencio A. (2020). Nasal Gene Expression of Angiotensin-Converting Enzyme 2 in Children and Adults. JAMA.

[35] Biswas S., Chakrabarti S. (2019). Increased Extracellular Matrix Protein Production in Chronic Diabetic Complications: Implications of Non-Coding RNAs. Noncoding RNA.

[36] Ma C.-X., Ma X.-N., Guan C.-H., Li Y.-D., Mauricio D., Fu S.-B. (2022). Cardiovascular disease in type 2 diabetes mellitus: Progress toward personalized management. Cardiovasc. Diabetol..

[37] Kyriakos G., Quiles-Sanchez L.V., Garmpi A., Farmaki P., Kyre K., Savvanis S., Antoniou V.K., Memi E. (2020). SGLT2 Inhibitors and Cardiovascular Outcomes: Do they Differ or there is a Class Effect? New Insights from the EMPA-REG OUTCOME trial and the CVD-REAL Study. Curr. Cardiol. Rev..

[38] Raciti G.A., Desiderio A., Longo M., Leone A., Zatterale F., Prevenzano I., Miele C., Napoli R., Beguinot F. (2021). DNA Methylation and Type 2 Diabetes: Novel Biomarkers for Risk Assessment?. Int. J. Mol. Sci..

[39] Dhawan S., Natarajan R. (2019). Epigenetics and Type 2 Diabetes Risk. Curr. Diabetes Rep..

[40] Baccarelli A., Ghosh S. (2012). Environmental exposures, epigenetics and cardiovascular disease. Curr. Opin. Clin. Nutr. Metab. Care.

[41] Hussain S., Khan A.W., Akhmedov A., Suades R., Costantino S., Paneni F., Caidahl K., Mohammed S.A., Hage C., Gkolfos C. (2020). Hyperglycemia Induces Myocardial Dysfunction via Epigenetic Regulation of JunD. Circ. Res..

[42] Deng X., Liu Y., Luo M., Wu J., Ma R., Wan Q., Wu J. (2017). Circulating miRNA-24 and its target YKL-40 as potential biomarkers in patients with coronary heart disease and type 2 diabetes mellitus. Oncotarget.

[43] Zhao T., Su Z., Li Y., Zhang X., You Q. (2020). Chitinase-3 like-protein-1 function and its role in diseases. Signal Transduct. Target. Ther..

[44] Kato M., Natarajan R. (2019). Epigenetics and epigenomics in diabetic kidney disease and metabolic memory. Nat. Rev. Nephrol..

[45] De Rosa S., Arcidiacono B., Chiefari E., Brunetti A., Indolfi C., Foti D.P. (2018). Type 2 Diabetes Mellitus and Cardiovascular Disease: Genetic and Epigenetic Links. Front. Endocrinol..

[46] Centers for Disease Control and Prevention Burden of Cigarette Use in the U.S. https://www.cdc.gov/tobacco/campaign/tips/resources/data/cigarette-smoking-in-united-states.html.

[47] Gallucci G., Tartarone A., Lerose R., Lalinga A.V., Capobianco A.M. (2020). Cardiovascular risk of smoking and benefits of smoking cessation. J. Thorac. Dis..

[48] Borgerding M., Klus H. (2005). Analysis of complex mixtures—Cigarette smoke. Exp. Toxicol. Pathol..

[49] Roy A., Rawal I., Jabbour S., Prabhakaran D., Prabhakaran D., Anand S., Gaziano T.A., Mbanya J.C., Wu Y., Nugent R. (2017). Tobacco and Cardiovascular Disease: A Summary of Evidence. Cardiovascular, Respiratory, and Related Disorders.

[50] Messner B., Bernhard D. (2014). Smoking and cardiovascular disease: Mechanisms of endothelial dysfunction and early atherogenesis. Arterioscler. Thromb. Vasc. Biol..

[51] Wu A.D., Lindson N., Hartmann-Boyce J., Wahedi A., Hajizadeh A., Theodoulou A., Thomas E.T., Lee C., Aveyard P. (2022). Smoking cessation for secondary prevention of cardiovascular disease. Cochrane Database Syst. Rev..

[52] Power C., Atherton K., Thomas C. (2010). Maternal smoking in pregnancy, adult adiposity and other risk factors for cardiovascular disease. Atherosclerosis.

[53] Bakker H., Jaddoe V.W. (2011). Cardiovascular and metabolic influences of fetal smoke exposure. Eur. J. Epidemiol..

[54] Ivashkiv L.B. (2018). IFNgamma: Signalling, epigenetics and roles in immunity, metabolism, disease and cancer immunotherapy. Nat. Rev. Immunol..

[55] Wang L., Li X., Zhou Y., Shi H., Xu C., He H., Wang S., Xiong X., Zhang Y., Du Z. (2014). Downregulation of miR-133 via MAPK/ERK signaling pathway involved in nicotine-induced cardiomyocyte apoptosis. Naunyn-Schmiedeberg’s Arch. Pharmacol..

[56] Fang F., Andersen A.M., Philibert R., Hancock D.B. (2023). Epigenetic biomarkers for smoking cessation. Addict. Neurosci..

[57] Centers for Disease Control and Prevention Facts About Hypertension. https://www.cdc.gov/bloodpressure/facts.htm.

[58] Kirkland E.B., Heincelman M., Bishu K.G., Schumann S.O., Schreiner A., Axon R.N., Mauldin P.D., Moran W.P. (2018). Trends in Healthcare Expenditures Among US Adults With Hypertension: National Estimates, 2003–2014. J. Am. Heart Assoc..

[59] Schutte A.E., Jafar T.H., Poulter N.R., Damasceno A., Khan N.A., Nilsson P.M., Alsaid J., Neupane D., Kario K., Beheiry H. (2023). Addressing global disparities in blood pressure control: Perspectives of the International Society of Hypertension. Cardiovasc. Res..

[60] Ferdinand K.C., Brown A.L. (2021). Will the 2021 USPSTF Hypertension Screening Recommendation Decrease or Worsen Racial/Ethnic Disparities in Blood Pressure Control?. JAMA Netw. Open.

[61] Stoll S., Wang C., Qiu H. (2018). DNA Methylation and Histone Modification in Hypertension. Int. J. Mol. Sci..

[62] Helmer A., Slater N., Smithgall S. (2018). A Review of ACE Inhibitors and ARBs in Black Patients With Hypertension. Ann. Pharmacother..

[63] Whelton P.K., Carey R.M., Aronow W.S., Casey D.E., Collins K.J., Dennison Himmelfarb C., DePalma S.M., Gidding S., Jamerson K.A., Jones D.W. (2018). 2017 ACC/AHA/AAPA/ABC/ACPM/AGS/APhA/ASH/ASPC/NMA/PCNA Guideline for the Prevention, Detection, Evaluation, and Management of High Blood Pressure in Adults: A Report of the American College of Cardiology/American Heart Association Task Force on Clinical Practice Guidelines. Hypertension.

[64] Richardson S.I., Freedman B.I., Ellison D.H., Rodriguez C.J. (2013). Salt sensitivity: A review with a focus on non-Hispanic blacks and Hispanics. J. Am. Soc. Hypertens..

[65] van der Linden E.L., Halley A., Meeks K.A.C., Chilunga F., Hayfron-Benjamin C., Venema A., Garrelds I.M., Danser A.H.J., van den Born B.-J., Henneman P. (2022). An explorative epigenome-wide association study of plasma renin and aldosterone concentration in a Ghanaian population: The RODAM study. Clin. Epigenet..

[66] Agha G., Mendelson M.M., Ward-Caviness C.K., Joehanes R., Huan T., Gondalia R., Salfati E., Brody J.A., Fiorito G., Bressler J. (2019). Blood Leukocyte DNA Methylation Predicts Risk of Future Myocardial Infarction and Coronary Heart Disease. Circulation.

[67] Chitrala K.N., Hernandez D.G., Nalls M.A., Mode N.A., Zonderman A.B., Ezike N., Evans M.K. (2020). Race-specific alterations in DNA methylation among middle-aged African Americans and Whites with metabolic syndrome. Epigenetics.

[68] Surendran P., Drenos F., Young R., Warren H., Cook J.P., Manning A.K., Grarup N., Sim X., Barnes D.R., Witkowska K. (2016). Trans-ancestry meta-analyses identify rare and common variants associated with blood pressure and hypertension. Nat. Genet..

[69] Liang M. (2018). Epigenetic Mechanisms and Hypertension. Hypertension.

[70] Suzuki K., Yamada H., Fujii R., Munetsuna E., Ando Y., Ohashi K., Ishikawa H., Yamazaki M., Maeda K., Hashimoto S. (2021). Association between circulating vascular-related microRNAs and an increase in blood pressure: A 5-year longitudinal population-based study. J. Hypertens..

[71] Choudhury M. (2022). Methods of Predicting Preeclampsia Using Biomarkers. U.S. Patent.

[72] Wu P., Haththotuwa R., Kwok C.S., Babu A., Kotronias R.A., Rushton C., Zaman A., Fryer A.A., Kadam U., Chew-Graham C.A. (2017). Preeclampsia and Future Cardiovascular Health. Circ. Cardiovasc. Qual. Outcomes.

[73] Hill M.F., Bordoni B. (2023). Hyperlipidemia. StatPearls.

[74] CDC (2023). National Hospital Ambulatory Medical Care Survey: 2019 National Summary Tables.

[75] U.S. Preventive Services Task Force (2022). Statin Use for the Primary Prevention of Cardiovascular Disease in Adults: US Preventive Services Task Force Recommendation Statement. JAMA.

[76] Bays H.E., Agarwala A., German C., Satish P., Iluyomade A., Dudum R., Thakkar A., Rifai M.A., Mehta A., Thobani A. (2022). Ten things to know about ten cardiovascular disease risk factors–2022. Am. J. Prev. Cardiol..

[77] Navar-Boggan A.M., Peterson E.D., D’Agostino R.B., Neely B., Sniderman A.D., Pencina M.J. (2015). Hyperlipidemia in early adulthood increases long-term risk of coronary heart disease. Circulation.

[78] Nelson R.H. (2013). Hyperlipidemia as a risk factor for cardiovascular disease. Prim. Care.

[79] Rafieian-Kopaei M., Setorki M., Doudi M., Baradaran A., Nasri H. (2014). Atherosclerosis: Process, indicators, risk factors and new hopes. Int. J. Prev. Med..

[80] Greißel A., Culmes M., Burgkart R., Zimmermann A., Eckstein H.-H., Zernecke A., Pelisek J. (2016). Histone acetylation and methylation significantly change with severity of atherosclerosis in human carotid plaques. Cardiovasc. Pathol..

[81] Zhu L., Jia L., Liu N., Wu R., Guan G., Hui R., Xing Y., Zhang Y., Wang J. (2022). DNA Methyltransferase 3b Accelerates the Process of Atherosclerosis. Oxid. Med. Cell. Longev..

[82] Kuan R., Agrawal D.K., Thankam F.G. (2021). Treg cells in atherosclerosis. Mol. Biol. Rep..

[83] Murray C.J., Atkinson C., Bhalla K., Birbeck G., Burstein R., Chou D., Dellavalle R., Danaei G., Ezzati M., Fahimi A. (2013). The state of US health, 1990-2010: Burden of diseases, injuries, and risk factors. JAMA.

[84] Pahwa R., Jialal I. (2023). Atherosclerosis. StatPearls.

[85] Wong N.D., Budoff M.J., Ferdinand K., Graham I.M., Michos E.D., Reddy T., Shapiro M.D., Toth P.P. (2022). Atherosclerotic cardiovascular disease risk assessment: An American Society for Preventive Cardiology clinical practice statement. Am. J. Prev. Cardiol..

[86] Anderson K.M., Wilson P.W., Odell P.M., Kannel W.B. (1991). An updated coronary risk profile. A statement for health professionals. Circulation.

[87] Okunrintemi V., Valero-Elizondo J., Patrick B., Salami J., Tibuakuu M., Ahmad S., Ogunmoroti O., Mahajan S., Khan S.U., Gulati M. (2018). Gender Differences in Patient-Reported Outcomes Among Adults With Atherosclerotic Cardiovascular Disease. J. Am. Heart Assoc..

[88] Lee M.T., Mahtta D., Ramsey D.J., Liu J., Misra A., Nasir K., Samad Z., Itchhaporia D., Khan S.U., Schofield R.S. (2021). Sex-Related Disparities in Cardiovascular Health Care Among Patients With Premature Atherosclerotic Cardiovascular Disease. JAMA Cardiol..

[89] Freaney P.M., Khan S.S., Lloyd-Jones D.M., Stone N.J. (2020). The Role of Sex-Specific Risk Factors in the Risk Assessment of Atherosclerotic Cardiovascular Disease for Primary Prevention in Women. Curr. Atheroscler. Rep..

[90] Rämö J.T., Ripatti P., Tabassum R., Söderlund S., Matikainen N., Gerl M.J., Klose C., Surma M.A., Stitziel N.O., Havulinna A.S. (2019). Coronary Artery Disease Risk and Lipidomic Profiles Are Similar in Hyperlipidemias With Family History and Population-Ascertained Hyperlipidemias. J. Am. Heart Assoc..

[91] Mihăilă R.G. (2020). Pragmatic Analysis of Dyslipidemia Involvement in Coronary Artery Disease: A Narrative Review. Curr. Cardiol. Rev..

[92] Khera A.V., Kathiresan S. (2017). Genetics of coronary artery disease: Discovery, biology and clinical translation. Nat. Rev. Genet..

[93] Kessler T., Schunkert H. (2021). Coronary Artery Disease Genetics Enlightened by Genome-Wide Association Studies. JACC Basic Transl. Sci..

[94] Poznyak A.V., Grechko A.V., Wetzker R., Orekhov A.N. (2020). In Search for Genes Related to Atherosclerosis and Dyslipidemia Using Animal Models. Int. J. Mol. Sci..

[95] Maneerat Y., Prasongsukarn K., Benjathummarak S., Dechkhajorn W. (2017). PPBP and DEFA1/DEFA3 genes in hyperlipidaemia as feasible synergistic inflammatory biomarkers for coronary heart disease. Lipids Health Dis..

[96] Eberharter A., Becker P.B. (2002). Histone acetylation: A switch between repressive and permissive chromatin. Second in review series on chromatin dynamics. EMBO Rep..

[97] Ho S.M., Johnson A., Tarapore P., Janakiram V., Zhang X., Leung Y.K. (2012). Environmental epigenetics and its implication on disease risk and health outcomes. Ilar J..

[98] Mazzio E.A., Soliman K.F. (2012). Basic concepts of epigenetics: Impact of environmental signals on gene expression. Epigenetics.

[99] Rendina González A.P., Preite V., Verhoeven K.J.F., Latzel V. (2018). Transgenerational Effects and Epigenetic Memory in the Clonal Plant Trifolium repens. Front. Plant Sci..

[100] de Castro Barbosa T., Ingerslev L.R., Alm P.S., Versteyhe S., Massart J., Rasmussen M., Donkin I., Sjögren R., Mudry J.M., Vetterli L. (2016). High-fat diet reprograms the epigenome of rat spermatozoa and transgenerationally affects metabolism of the offspring. Mol. Metab..

[101] Heijmans B.T., Tobi E.W., Stein A.D., Putter H., Blauw G.J., Susser E.S., Slagboom P.E., Lumey L.H. (2008). Persistent epigenetic differences associated with prenatal exposure to famine in humans. Proc. Natl. Acad. Sci. USA.

[102] Bleker L.S., de Rooij S.R., Painter R.C., Ravelli A.C., Roseboom T.J. (2021). Cohort profile: The Dutch famine birth cohort (DFBC)- a prospective birth cohort study in the Netherlands. BMJ Open.

[103] Abraham M.J., El Sherbini A., El-Diasty M., Askari S., Szewczuk M.R. (2023). Restoring Epigenetic Reprogramming with Diet and Exercise to Improve Health-Related Metabolic Diseases. Biomolecules.

[104] Almodóvar A.S., Nahata M.C. (2019). Associations Between Chronic Disease, Polypharmacy, and Medication-Related Problems Among Medicare Beneficiaries. J. Manag. Care Spec. Pharm..

[105] Sheikh-Taha M., Asmar M. (2021). Polypharmacy and severe potential drug-drug interactions among older adults with cardiovascular disease in the United States. BMC Geriatr..

[106] Zhao M., Liu C.F., Feng Y.F., Chen H. (2022). Potential drug-drug interactions in drug therapy for older adults with chronic coronary syndrome at hospital discharge: A real-world study. Front. Pharmacol..

[107] Laslett D.B., Cooper J.M., Greenberg R.M., Yesenosky G.A., Basil A., Gangireddy C., Whitman I.R. (2020). Electrolyte Abnormalities in Patients Presenting With Ventricular Arrhythmia (from the LYTE-VT Study). Am. J. Cardiol..

[108] Skogestad J., Aronsen J.M. (2018). Hypokalemia-Induced Arrhythmias and Heart Failure: New Insights and Implications for Therapy. Front. Physiol..

[109] van der Laan D.M., Elders P.J.M., Boons C., Nijpels G., Krska J., Hugtenburg J.G. (2018). The impact of cardiovascular medication use on patients’ daily lives: A cross-sectional study. Int. J. Clin. Pharm..

[110] Leslie K.H., McCowan C., Pell J.P. (2019). Adherence to cardiovascular medication: A review of systematic reviews. J. Public Health.

[111] Bansal A.B., Cassagnol M. (2023). HMG-CoA Reductase Inhibitors. StatPearls.

[112] Diamantis E., Kyriakos G., Quiles-Sanchez L.V., Farmaki P., Troupis T. (2017). The Anti-Inflammatory Effects of Statins on Coronary Artery Disease: An Updated Review of the Literature. Curr. Cardiol. Rev..

[113] Arnett D.K., Blumenthal R.S., Albert M.A., Buroker A.B., Goldberger Z.D., Hahn E.J., Himmelfarb C.D., Khera A., Lloyd-Jones D., McEvoy J.W. (2019). 2019 ACC/AHA Guideline on the Primary Prevention of Cardiovascular Disease: Executive Summary: A Report of the American College of Cardiology/American Heart Association Task Force on Clinical Practice Guidelines. Circulation.

[114] Tarn D.M., Pletcher M.J., Tosqui R., Fernandez A., Tseng C.-h., Moriconi R., Bell D.S., Barrientos M., Turner J.A., Schwartz J.B. (2021). Primary nonadherence to statin medications: Survey of patient perspectives. Prev. Med. Rep..

[115] Lavie G., Hoshen M., Leibowitz M., Benis A., Akriv A., Balicer R., Reges O. (2021). Statin Therapy for Primary Prevention in the Elderly and Its Association with New-Onset Diabetes, Cardiovascular Events, and All-Cause Mortality. Am. J. Med..

[116] Tangelloju S., Little B.B., Esterhay R.J., Brock G., LaJoie S. (2020). Statins are associated with new onset type 2 diabetes mellitus (T2DM) in Medicare patients ≥65 years. Diabetes Metab. Res. Rev..

[117] Singh H., Sikarwar P., Khurana S., Sharma J. (2022). Assessing the Incidence of New-onset Diabetes Mellitus with Statin Use: A Systematic Review of the Systematic Reviews and Meta-analyses. Touchrev Endocrinol..

[118] Mollazadeh H., Tavana E., Fanni G., Bo S., Banach M., Pirro M., von Haehling S., Jamialahmadi T., Sahebkar A. (2021). Effects of statins on mitochondrial pathways. J. Cachexia Sarcopenia Muscle.

[119] Tricarico P.M., Crovella S., Celsi F. (2015). Mevalonate Pathway Blockade, Mitochondrial Dysfunction and Autophagy: A Possible Link. Int. J. Mol. Sci..

[120] Galicia-Garcia U., Jebari S., Larrea-Sebal A., Uribe K.B., Siddiqi H., Ostolaza H., Benito-Vicente A., Martín C. (2020). Statin Treatment-Induced Development of Type 2 Diabetes: From Clinical Evidence to Mechanistic Insights. Int. J. Mol. Sci..

[121] Lin Y.-C., Lin J.-H., Chou C.-W., Chang Y.-F., Yeh S.-H., Chen C.-C. (2008). Statins Increase p21 through Inhibition of Histone Deacetylase Activity and Release of Promoter-Associated HDAC1/2. Cancer Res..

[122] Yoon S., Eom G.H. (2016). HDAC and HDAC Inhibitor: From Cancer to Cardiovascular Diseases. Chonnam Med. J..

[123] Gillette T.G. (2021). HDAC Inhibition in the Heart. Circulation.

[124] Wang Z., Zhao Y.T., Zhao T.C. (2021). Histone deacetylases in modulating cardiac disease and their clinical translational and therapeutic implications. Exp. Biol. Med..

[125] Andolina D., Savi M., Ielpo D., Barbetti M., Bocchi L., Stilli D., Ventura R., Lo Iacono L., Sgoifo A., Carnevali L. (2021). Elevated miR-34a expression and altered transcriptional profile are associated with adverse electromechanical remodeling in the heart of male rats exposed to social stress. Stress.

[126] Han H., Qu G., Han C., Wang Y., Sun T., Li F., Wang J., Luo S. (2015). MiR-34a, miR-21 and miR-23a as potential biomarkers for coronary artery disease: A pilot microarray study and confirmation in a 32 patient cohort. Exp. Mol. Med..

[127] Gatsiou A., Georgiopoulos G., Vlachogiannis N.I., Pfisterer L., Fischer A., Sachse M., Laina A., Bonini F., Delialis D., Tual-Chalot S. (2021). Additive contribution of microRNA-34a/b/c to human arterial ageing and atherosclerosis. Atherosclerosis.

[128] Hua C.C., Liu X.M., Liang L.R., Wang L.F., Zhong J.C. (2021). Targeting the microRNA-34a as a Novel Therapeutic Strategy for Cardiovascular Diseases. Front. Cardiovasc. Med..

[129] Raucci A., Macrì F., Castiglione S., Badi I., Vinci M.C., Zuccolo E. (2021). MicroRNA-34a: The bad guy in age-related vascular diseases. Cell. Mol. Life Sci..

[130] Tabuchi T., Satoh M., Itoh T., Nakamura M. (2012). MicroRNA-34a regulates the longevity-associated protein SIRT1 in coronary artery disease: Effect of statins on SIRT1 and microRNA-34a expression. Clin. Sci..

[131] Karlic H., Thaler R., Gerner C., Grunt T., Proestling K., Haider F., Varga F. (2015). Inhibition of the mevalonate pathway affects epigenetic regulation in cancer cells. Cancer Genet..

[132] Weber M.A., Schiffrin E.L., White W.B., Mann S., Lindholm L.H., Kenerson J.G., Flack J.M., Carter B.L., Materson B.J., Ram C.V. (2014). Clinical practice guidelines for the management of hypertension in the community: A statement by the American Society of Hypertension and the International Society of Hypertension. J. Clin. Hypertens..

[133] Basile J. (2004). The role of existing and newer calcium channel blockers in the treatment of hypertension. J. Clin. Hypertens..

[134] McKeever R.G., Hamilton R.J. (2023). Calcium Channel Blockers. StatPearls.

[135] Godfraind T. (2014). Calcium Channel Blockers in Cardiovascular Pharmacotherapy. J. Cardiovasc. Pharmacol. Ther..

[136] Sueta D., Tabata N., Hokimoto S. (2017). Clinical roles of calcium channel blockers in ischemic heart diseases. Hypertens. Res..

[137] Aronow W.S. (2019). Medical management after myocardial infarction. Future Cardiol..

[138] Heidenreich P.A., Bozkurt B., Aguilar D., Allen L.A., Byun J.J., Colvin M.M., Deswal A., Drazner M.H., Dunlay S.M., Evers L.R. (2022). 2022 AHA/ACC/HFSA Guideline for the Management of Heart Failure: A Report of the American College of Cardiology/American Heart Association Joint Committee on Clinical Practice Guidelines. Circulation.

[139] Palandri C., Santini L., Argirò A., Margara F., Doste R., Bueno-Orovio A., Olivotto I., Coppini R. (2022). Pharmacological Management of Hypertrophic Cardiomyopathy: From Bench to Bedside. Drugs.

[140] Leitch J.W., McElduff P., Dobson A., Heller R. (1998). Outcome With Calcium Channel Antagonists After Myocardial Infarction: A Community-Based Study. J. Am. Coll. Cardiol..

[141] Thakur A.A., Wang X., Garcia-Betancourt M.M., Forse R.A. (2018). Calcium channel blockers and the incidence of breast and prostate cancer: A meta-analysis. J. Clin. Pharm. Ther..

[142] Saltzman B.S., Weiss N.S., Sieh W., Fitzpatrick A.L., McTiernan A., Daling J.R., Li C.I. (2013). Use of antihypertensive medications and breast cancer risk. Cancer Causes Control.

[143] Rotshild V., Hirsh Raccah B., Gazawe M., Matok I. (2022). Calcium Channel Blocker Use and the Risk for Breast Cancer: A Population-Based Nested Case-Control Study. Cancers.

[144] Kizer J.R., Kimmel S.E. (2001). Epidemiologic review of the calcium channel blocker drugs. An up-to-date perspective on the proposed hazards. Arch. Intern. Med..

[145] Elliott W.J., Ram C.V.S. (2011). Calcium Channel Blockers. J. Clin. Hypertens..

[146] Tang B., Li X., Wang Y., Sjölander A., Johnell K., Thambisetty M., Ferrucci L., Reynolds C.A., Finkel D., Jylhävä J. (2023). Longitudinal associations between use of antihypertensive, antidiabetic, and lipid-lowering medications and biological aging. GeroScience.

[147] Kho M., Wang Y.Z., Chaar D., Zhao W., Ratliff S.M., Mosley T.H., Peyser P.A., Kardia S.L.R., Smith J.A. (2021). Accelerated DNA methylation age and medication use among African Americans. Aging.

[148] Dai H., Sinclair D.A., Ellis J.L., Steegborn C. (2018). Sirtuin activators and inhibitors: Promises, achievements, and challenges. Pharmacol. Ther..

[149] Manna D., Bhuyan R., Ghosh R. (2018). Probing the mechanism of SIRT1 activation by a 1,4-dihydropyridine. J. Mol. Model..

[150] Zwergel C., Aventaggiato M., Garbo S., Di Bello E., Fassari B., Noce B., Castiello C., Lambona C., Barreca F., Rotili D. (2023). Novel 1,4-Dihydropyridines as Specific Binders and Activators of SIRT3 Impair Cell Viability and Clonogenicity and Downregulate Hypoxia-Induced Targets in Cancer Cells. J. Med. Chem..

[151] Holley A.K., Bakthavatchalu V., Velez-Roman J.M., St Clair D.K. (2011). Manganese superoxide dismutase: Guardian of the powerhouse. Int. J. Mol. Sci..

[152] Kane A.E., Sinclair D.A. (2018). Sirtuins and NAD+ in the Development and Treatment of Metabolic and Cardiovascular Diseases. Circ. Res..

[153] Matsushima S., Sadoshima J. (2015). The role of sirtuins in cardiac disease. Am. J. Physiol. Heart Circ. Physiol..

[154] Farzam K., Jan A. (2023). Beta Blockers. StatPearls.

[155] Gorre F., Vandekerckhove H. (2010). Beta-blockers: Focus on mechanism of action Which beta-blocker, when and why?. Acta Cardiol..

[156] Schena G., Caplan M.J. (2019). Everything You Always Wanted to Know about β(3)-AR * (* But Were Afraid to Ask). Cells.

[157] Divan I., Suleman A., Lars L., Joakim A., Tatendashe Bernadette D., Claes H., Tomas J., Troels Y., Chris P.G., Gorav B. (2023). Association of beta-blockers beyond 1 year after myocardial infarction and cardiovascular outcomes. Heart.

[158] Godoy L.C., Farkouh M.E., Austin P.C., Shah B.R., Qiu F., Jackevicius C.A., Wijeysundera H.C., Krumholz H.M., Ko D.T. (2023). Association of Beta-Blocker Therapy With Cardiovascular Outcomes in Patients With Stable Ischemic Heart Disease. J. Am. Coll. Cardiol..

[159] Bangalore S., Steg G., Deedwania P., Crowley K., Eagle K.A., Goto S., Ohman E.M., Cannon C.P., Smith S.C., Zeymer U. (2012). β-Blocker Use and Clinical Outcomes in Stable Outpatients With and Without Coronary Artery Disease. JAMA.

[160] Ziff O.J., Samra M., Howard J.P., Bromage D.I., Ruschitzka F., Francis D.P., Kotecha D. (2020). Beta-blocker efficacy across different cardiovascular indications: An umbrella review and meta-analytic assessment. BMC Med..

[161] Dézsi C.A., Szentes V. (2017). The Real Role of β-Blockers in Daily Cardiovascular Therapy. Am. J. Cardiovasc. Drugs.

[162] Barron A.J., Zaman N., Cole G.D., Wensel R., Okonko D.O., Francis D.P. (2013). Systematic review of genuine versus spurious side-effects of beta-blockers in heart failure using placebo control: Recommendations for patient information. Int. J. Cardiol..

[163] Lin W., Huang Z., Ping S., Zhang S., Wen X., He Y., Ren Y. (2022). Toxicological effects of atenolol and venlafaxine on zebrafish tissues: Bioaccumulation, DNA hypomethylation, and molecular mechanism. Environ. Pollut..

[164] Napoli C., Grimaldi V., De Pascale M.R., Sommese L., Infante T., Soricelli A. (2016). Novel epigenetic-based therapies useful in cardiovascular medicine. World J. Cardiol..

[165] Zhong J., Agha G., Baccarelli A.A. (2016). The Role of DNA Methylation in Cardiovascular Risk and Disease: Methodological Aspects, Study Design, and Data Analysis for Epidemiological Studies. Circ. Res..

[166] Solayman M.H., Langaee T.Y., Gong Y., Shahin M.H., Turner S.T., Chapman A.B., Gums J.G., Boerwinkle E., Beitelshees A.L., El-Hamamsy M. (2019). Effect of plasma MicroRNA on antihypertensive response to beta blockers in the Pharmacogenomic Evaluation of Antihypertensive Responses (PEAR) studies. Eur. J. Pharm. Sci..

[167] Kovell L.C., Ahmed H.M., Misra S., Whelton S.P., Prokopowicz G.P., Blumenthal R.S., McEvoy J.W. (2015). US Hypertension Management Guidelines: A Review of the Recent Past and Recommendations for the Future. J. Am. Heart Assoc..

[168] Tai C., Gan T., Zou L., Sun Y., Zhang Y., Chen W., Li J., Zhang J., Xu Y., Lu H. (2017). Effect of angiotensin-converting enzyme inhibitors and angiotensin II receptor blockers on cardiovascular events in patients with heart failure: A meta-analysis of randomized controlled trials. BMC Cardiovasc. Disord..

[169] Messerli Franz H., Bangalore S., Bavishi C., Rimoldi Stefano F. (2018). Angiotensin-Converting Enzyme Inhibitors in Hypertension. J. Am. Coll. Cardiol..

[170] Awad K., Zaki M.M., Mohammed M., Lewek J., Lavie C.J., Banach M. (2022). Effect of the Renin-Angiotensin System Inhibitors on Inflammatory Markers: A Systematic Review and Meta-analysis of Randomized Controlled Trials. Mayo Clin. Proc..

[171] Herman L.L., Padala S.A., Ahmed I., Bashir K. (2023). Angiotensin-Converting Enzyme Inhibitors (ACEI). StatPearls.

[172] Yılmaz İ. (2019). Angiotensin-Converting Enzyme Inhibitors Induce Cough. Turk. Thorac. J..

[173] Pinto B., Jadhav U., Singhai P., Sadhanandham S., Shah N. (2020). ACEI-induced cough: A review of current evidence and its practical implications for optimal CV risk reduction. Indian Heart J..

[174] Mirabito Colafella K.M., Uijl E., Jan Danser A.H., Huhtaniemi I., Martini L. (2019). Interference with the Renin–Angiotensin System (RAS): Classical Inhibitors and Novel Approaches. Encyclopedia of Endocrine Diseases.

[175] Burnier M. (2001). Angiotensin II Type 1 Receptor Blockers. Circulation.

[176] Barreras A., Gurk-Turner C. (2003). Angiotensin II receptor blockers. Proc. (Bayl. Univ. Med. Cent.).

[177] Reddy M.A., Sumanth P., Lanting L., Yuan H., Wang M., Mar D., Alpers C.E., Bomsztyk K., Natarajan R. (2014). Losartan reverses permissive epigenetic changes in renal glomeruli of diabetic db/db mice. Kidney Int..

[178] Wang T., Lian G., Cai X., Lin Z., Xie L. (2018). Effect of prehypertensive losartan therapy on AT1R and ATRAP methylation of adipose tissue in the later life of high-fat-fed spontaneously hypertensive rats. Mol. Med. Rep..

[179] Seto E., Yoshida M. (2014). Erasers of histone acetylation: The histone deacetylase enzymes. Cold Spring Harb. Perspect. Biol..

[180] Park S.-Y., Kim J.-S. (2020). A short guide to histone deacetylases including recent progress on class II enzymes. Exp. Mol. Med..

[181] Li G., Tian Y., Zhu W.-G. (2020). The Roles of Histone Deacetylases and Their Inhibitors in Cancer Therapy. Front. Cell Dev. Biol..

[182] Bagchi R.A., Weeks K.L. (2019). Histone deacetylases in cardiovascular and metabolic diseases. J. Mol. Cell. Cardiol..

[183] Lee H.T., Oh S., Ro D.H., Yoo H., Kwon Y.W. (2020). The Key Role of DNA Methylation and Histone Acetylation in Epigenetics of Atherosclerosis. J. Lipid Atheroscler..

[184] Kronlage M., Katus H.A., Backs J., Backs J., McKinsey T.A. (2016). HDAC Signaling Networks in Heart Failure. Epigenetics in Cardiac Disease.

[185] Kabra D.G., Pfuhlmann K., García-Cáceres C., Schriever S.C., Casquero García V., Kebede A.F., Fuente-Martin E., Trivedi C., Heppner K., Uhlenhaut N.H. (2016). Hypothalamic leptin action is mediated by histone deacetylase 5. Nat. Commun..

[186] Çakır I., Hadley C.K., Pan P.L., Bagchi R.A., Ghamari-Langroudi M., Porter D.T., Wang Q., Litt M.J., Jana S., Hagen S. (2022). Histone deacetylase 6 inhibition restores leptin sensitivity and reduces obesity. Nat. Metab..

[187] Chen Y., Du J., Zhao Y.T., Zhang L., Lv G., Zhuang S., Qin G., Zhao T.C. (2015). Histone deacetylase (HDAC) inhibition improves myocardial function and prevents cardiac remodeling in diabetic mice. Cardiovasc. Diabetol..

[188] Jung J.K., Yoon G.-E., Jang G., Park K.M., Kim I., Kim J.I. (2021). Inhibition of HDACs (Histone Deacetylases) Ameliorates High-Fat Diet–Induced Hypertension Through Restoration of the MsrA (Methionine Sulfoxide Reductase A)/Hydrogen Sulfide Axis. Hypertension.

[189] Rahman M., Awosika A.O., Nguyen H. (2023). Valproic Acid. StatPearls.

[190] Göttlicher M., Minucci S., Zhu P., Krämer O.H., Schimpf A., Giavara S., Sleeman J.P., Lo Coco F., Nervi C., Pelicci P.G. (2001). Valproic acid defines a novel class of HDAC inhibitors inducing differentiation of transformed cells. Embo J..

[191] Sixto-López Y., Bello M., Correa-Basurto J. (2020). Exploring the inhibitory activity of valproic acid against the HDAC family using an MMGBSA approach. J. Comput.-Aided Mol. Des..

[192] Lee H.-A., Lee D.-Y., Cho H.-M., Kim S.-Y., Iwasaki Y., Kim I.K. (2013). Histone Deacetylase Inhibition Attenuates Transcriptional Activity of Mineralocorticoid Receptor Through Its Acetylation and Prevents Development of Hypertension. Circ. Res..

[193] Terker A.S., Ellison D.H. (2015). Renal mineralocorticoid receptor and electrolyte homeostasis. Am. J. Physiol. Regul. Integr. Comp. Physiol..

[194] Scholz B., Schulte J.S., Hamer S., Himmler K., Pluteanu F., Seidl M.D., Stein J., Wardelmann E., Hammer E., Völker U. (2019). HDAC (Histone Deacetylase) Inhibitor Valproic Acid Attenuates Atrial Remodeling and Delays the Onset of Atrial Fibrillation in Mice. Circ. Arrhythmia Electrophysiol..

[195] Odutayo A., Wong C.X., Hsiao A.J., Hopewell S., Altman D.G., Emdin C.A. (2016). Atrial fibrillation and risks of cardiovascular disease, renal disease, and death: Systematic review and meta-analysis. BMJ.

[196] Tian S., Lei I., Gao W., Liu L., Guo Y., Creech J., Herron T.J., Xian S., Ma P.X., Eugene Chen Y. (2019). HDAC inhibitor valproic acid protects heart function through Foxm1 pathway after acute myocardial infarction. EBioMedicine.

[197] English J.D., Tian S., Wang Z., Luzum J.A. (2022). Association of Valproic Acid Use With Post-Myocardial Infarction Heart Failure Development: A Meta-Analysis of Two Retrospective Case-Control Studies. J. Cardiovasc. Pharmacol. Ther..

[198] Kusaczuk M., Krętowski R., Bartoszewicz M., Cechowska-Pasko M. (2016). Phenylbutyrate-a pan-HDAC inhibitor-suppresses proliferation of glioblastoma LN-229 cell line. Tumour Biol..

[199] Daosukho C., Chen Y., Noel T., Sompol P., Nithipongvanitch R., Velez J.M., Oberley T.D., St Clair D.K. (2007). Phenylbutyrate, a histone deacetylase inhibitor, protects against Adriamycin-induced cardiac injury. Free Radic. Biol. Med..

[200] Takatori O., Usui S., Okajima M., Kaneko S., Ootsuji H., Takashima S.-i., Kobayashi D., Murai H., Furusho H., Takamura M. (2016). Sodium 4-Phenylbutyrate Attenuates Myocardial Reperfusion Injury by Reducing the Unfolded Protein Response. J. Cardiovasc. Pharmacol. Ther..

[201] Wu Y., Adi D., Long M., Wang J., Liu F., Gai M.T., Aierken A., Li M.Y., Li Q., Wu L.Q. (2016). 4-Phenylbutyric Acid Induces Protection against Pulmonary Arterial Hypertension in Rats. PLoS ONE.

[202] Ma J., Luo T., Zeng Z., Fu H., Asano Y., Liao Y., Minamino T., Kitakaze M. (2016). Histone Deacetylase Inhibitor Phenylbutyrate Exaggerates Heart Failure in Pressure Overloaded Mice independently of HDAC inhibition. Sci. Rep..

[203] Bubna A.K. (2015). Vorinostat-An Overview. Indian J. Dermatol..

[204] Pedro Ferreira J., Pitt B., Zannad F. (2021). Histone deacetylase inhibitors for cardiovascular conditions and healthy longevity. Lancet Healthy Longev..

[205] Xie M., Kong Y., Tan W., May H., Battiprolu P.K., Pedrozo Z., Wang Z.V., Morales C., Luo X., Cho G. (2014). Histone deacetylase inhibition blunts ischemia/reperfusion injury by inducing cardiomyocyte autophagy. Circulation.

[206] Nagata S., Marunouchi T., Tanonaka K. (2019). Histone Deacetylase Inhibitor SAHA Treatment Prevents the Development of Heart Failure after Myocardial Infarction *via* an Induction of Heat-Shock Proteins in Rats. Biol. Pharm. Bull..

[207] Patnaik S., Nathan S., Kar B., Gregoric I.D., Li Y.-P. (2023). The Role of Extracellular Heat Shock Proteins in Cardiovascular Diseases. Biomedicines.

[208] Chelladurai P., Dabral S., Basineni S.R., Chen C.-N., Schmoranzer M., Bender N., Feld C., Nötzold R.R., Dobreva G., Wilhelm J. (2020). Isoform-specific characterization of class I histone deacetylases and their therapeutic modulation in pulmonary hypertension. Sci. Rep..

[209] Ma J., Guo X., Zhang S., Liu H., Lu J., Dong Z., Liu K., Ming L. (2015). Trichostatin A, a histone deacetylase inhibitor, suppresses proliferation and promotes apoptosis of esophageal squamous cell lines. Mol. Med. Rep..

[210] Vigushin D.M., Ali S., Pace P.E., Mirsaidi N., Ito K., Adcock I., Coombes R.C. (2001). Trichostatin A Is a Histone Deacetylase Inhibitor with Potent Antitumor Activity against Breast Cancer in Vivo1. Clin. Cancer Res..

[211] Muslin A.J. (2008). MAPK signalling in cardiovascular health and disease: Molecular mechanisms and therapeutic targets. Clin. Sci..

[212] Zhang W., Elimban V., Nijjar M.S., Gupta S.K., Dhalla N.S. (2003). Role of mitogen-activated protein kinase in cardiac hypertrophy and heart failure. Exp. Clin. Cardiol..

[213] Yang D., Xie P., Liu Z. (2012). Ischemia/reperfusion-induced MKP-3 impairs endothelial NO formation via inactivation of ERK1/2 pathway. PLoS ONE.

[214] Tran N., Garcia T., Aniqa M., Ali S., Ally A., Nauli S.M. (2022). Endothelial Nitric Oxide Synthase (eNOS) and the Cardiovascular System: In Physiology and in Disease States. Am. J. Biomed. Sci. Res..

[215] Turner N.A., Blythe N.M. (2019). Cardiac Fibroblast p38 MAPK: A Critical Regulator of Myocardial Remodeling. J. Cardiovasc. Dev. Dis..

[216] Somanna N.K., Valente A.J., Krenz M., McDonald K.S., Higashi Y., Noda M., Chandrasekar B. (2016). Histone deacetyltransferase inhibitors Trichostatin A and Mocetinostat differentially regulate MMP9, IL-18 and RECK expression, and attenuate Angiotensin II-induced cardiac fibroblast migration and proliferation. Hypertens. Res..

[217] Houtkooper R.H., Pirinen E., Auwerx J. (2012). Sirtuins as regulators of metabolism and healthspan. Nat. Rev. Mol. Cell Biol..

[218] Vassilopoulos A., Fritz K.S., Petersen D.R., Gius D. (2011). The human sirtuin family: Evolutionary divergences and functions. Hum. Genom..

[219] Bindu S., Pillai V.B., Gupta M.P. (2016). Role of Sirtuins in Regulating Pathophysiology of the Heart. Trends Endocrinol. Metab..

[220] Carafa V., Rotili D., Forgione M., Cuomo F., Serretiello E., Hailu G.S., Jarho E., Lahtela-Kakkonen M., Mai A., Altucci L. (2016). Sirtuin functions and modulation: From chemistry to the clinic. Clin. Epigenet..

[221] Salehi B., Mishra A.P., Nigam M., Sener B., Kilic M., Sharifi-Rad M., Fokou P.V.T., Martins N., Sharifi-Rad J. (2018). Resveratrol: A Double-Edged Sword in Health Benefits. Biomedicines.

[222] Michno A., Grużewska K., Ronowska A., Gul-Hinc S., Zyśk M., Jankowska-Kulawy A. (2022). Resveratrol Inhibits Metabolism and Affects Blood Platelet Function in Type 2 Diabetes. Nutrients.

[223] Zheng X., Hai J., Yang Y., Zhang C., Ma X., Kong B., Zhao Y., Hu Y., Bu P., Ti Y. (2023). Effects of resveratrol supplementation on cardiac remodeling in hypertensive patients: A randomized controlled clinical trial. Hypertens. Res..

[224] Kuro-o M. (2019). The Klotho proteins in health and disease. Nat. Rev. Nephrol..

[225] Zhang J., Xiang H., Liu J., Chen Y., He R.R., Liu B. (2020). Mitochondrial Sirtuin 3: New emerging biological function and therapeutic target. Theranostics.

[226] Younus H. (2018). Therapeutic potentials of superoxide dismutase. Int. J. Health Sci..

[227] Mathieu L., Lopes Costa A., Le Bachelier C., Slama A., Lebre A.-S., Taylor R.W., Bastin J., Djouadi F. (2016). Resveratrol attenuates oxidative stress in mitochondrial Complex I deficiency: Involvement of SIRT3. Free Radic. Biol. Med..

[228] Dyck G.J.B., Raj P., Zieroth S., Dyck J.R.B., Ezekowitz J.A. (2019). The Effects of Resveratrol in Patients with Cardiovascular Disease and Heart Failure: A Narrative Review. Int. J. Mol. Sci..

[229] Mao Q., Liang X., Wu Y., Lu Y. (2019). Resveratrol Attenuates Cardiomyocyte Apoptosis in Rats Induced by Coronary Microembolization Through SIRT1-Mediated Deacetylation of p53. J. Cardiovasc. Pharmacol. Ther..

[230] Ma S., Feng J., Zhang R., Chen J., Han D., Li X., Yang B., Li X., Fan M., Li C. (2017). SIRT1 Activation by Resveratrol Alleviates Cardiac Dysfunction via Mitochondrial Regulation in Diabetic Cardiomyopathy Mice. Oxid. Med. Cell. Longev..

[231] Marmorstein R. (2001). Structure and function of histone acetyltransferases. Cell. Mol. Life Sci..

[232] Lee K.K., Workman J.L. (2007). Histone acetyltransferase complexes: One size doesn’t fit all. Nat. Rev. Mol. Cell Biol..

[233] Costantino S., Libby P., Kishore R., Tardif J.-C., El-Osta A., Paneni F. (2018). Epigenetics and precision medicine in cardiovascular patients: From basic concepts to the clinical arena. Eur. Heart J..

[234] Wang Y., Miao X., Liu Y., Li F., Liu Q., Sun J., Cai L. (2014). Dysregulation of histone acetyltransferases and deacetylases in cardiovascular diseases. Oxid. Med. Cell. Longev..

[235] Yang M., Zhang Y., Ren J. (2020). Acetylation in cardiovascular diseases: Molecular mechanisms and clinical implications. Biochim. Biophys. Acta (BBA)-Mol. Basis Dis..

[236] Nelson K.M., Dahlin J.L., Bisson J., Graham J., Pauli G.F., Walters M.A. (2017). The Essential Medicinal Chemistry of Curcumin. J. Med. Chem..

[237] Balasubramanyam K., Varier R.A., Altaf M., Swaminathan V., Siddappa N.B., Ranga U., Kundu T.K. (2004). Curcumin, a Novel p300/CREB-binding Protein-specific Inhibitor of Acetyltransferase, Represses the Acetylation of Histone/Nonhistone Proteins and Histone Acetyltransferase-dependent Chromatin Transcription*. J. Biol. Chem..

[238] Marcu M.G., Jung Y.J., Lee S., Chung E.J., Lee M.J., Trepel J., Neckers L. (2006). Curcumin is an inhibitor of p300 histone acetylatransferase. Med. Chem..

[239] Hassan F.-u., Rehman M.S.-u., Khan M.S., Ali M.A., Javed A., Nawaz A., Yang C. (2019). Curcumin as an Alternative Epigenetic Modulator: Mechanism of Action and Potential Effects. Front. Genet..

[240] Morimoto T., Sunagawa Y., Kawamura T., Takaya T., Wada H., Nagasawa A., Komeda M., Fujita M., Shimatsu A., Kita T. (2008). The dietary compound curcumin inhibits p300 histone acetyltransferase activity and prevents heart failure in rats. J. Clin. Investig..

[241] Sunagawa Y., Funamoto M., Shimizu K., Shimizu S., Sari N., Katanasaka Y., Miyazaki Y., Kakeya H., Hasegawa K., Morimoto T. (2021). Curcumin, an Inhibitor of p300-HAT Activity, Suppresses the Development of Hypertension-Induced Left Ventricular Hypertrophy with Preserved Ejection Fraction in Dahl Rats. Nutrients.

[242] Välimäki M.J., Ruskoaho H.J. (2020). Targeting GATA4 for cardiac repair. IUBMB Life.

[243] Zhao J., Chen Y., Chen Q., Hong T., Zhong Z., He J., Ni C. (2022). Curcumin Ameliorates Cardiac Fibrosis by Regulating Macrophage-Fibroblast Crosstalk via IL18-P-SMAD2/3 Signaling Pathway Inhibition. Front. Pharmacol..

[244] Li C., Meng X., Wang L., Dai X. (2023). Mechanism of action of non-coding RNAs and traditional Chinese medicine in myocardial fibrosis: Focus on the TGF-β/Smad signaling pathway. Front. Pharmacol..

[245] Qin S., Huang L., Gong J., Shen S., Huang J., Ren H., Hu H. (2017). Efficacy and safety of turmeric and curcumin in lowering blood lipid levels in patients with cardiovascular risk factors: A meta-analysis of randomized controlled trials. Nutr. J..

[246] Tomé-Carneiro J., Gonzálvez M., Larrosa M., García-Almagro F.J., Avilés-Plaza F., Parra S., Yáñez-Gascón M.J., Ruiz-Ros J.A., García-Conesa M.T., Tomás-Barberán F.A. (2012). Consumption of a grape extract supplement containing resveratrol decreases oxidized LDL and ApoB in patients undergoing primary prevention of cardiovascular disease: A triple-blind, 6-month follow-up, placebo-controlled, randomized trial. Mol. Nutr. Food Res..

[247] Tomé-Carneiro J., Larrosa M., Yáñez-Gascón M.J., Dávalos A., Gil-Zamorano J., Gonzálvez M., García-Almagro F.J., Ruiz Ros J.A., Tomás-Barberán F.A., Espín J.C. (2013). One-year supplementation with a grape extract containing resveratrol modulates inflammatory-related microRNAs and cytokines expression in peripheral blood mononuclear cells of type 2 diabetes and hypertensive patients with coronary artery disease. Pharmacol. Res..

[248] Tomé-Carneiro J., Gonzálvez M., Larrosa M., Yáñez-Gascón M.J., García-Almagro F.J., Ruiz-Ros J.A., Tomás-Barberán F.A., García-Conesa M.T., Espín J.C. (2013). Grape Resveratrol Increases Serum Adiponectin and Downregulates Inflammatory Genes in Peripheral Blood Mononuclear Cells: A Triple-Blind, Placebo-Controlled, One-Year Clinical Trial in Patients with Stable Coronary Artery Disease. Cardiovasc. Drugs Ther..

[249] Garg A.X., Devereaux P.J., Hill A., Sood M., Aggarwal B., Dubois L., Hiremath S., Guzman R., Iyer V., James M. (2018). Oral curcumin in elective abdominal aortic aneurysm repair: A multicentre randomized controlled trial. Can. Med. Assoc. J..

[250] Wongcharoen W., Jai-Aue S., Phrommintikul A., Nawarawong W., Woragidpoonpol S., Tepsuwan T., Sukonthasarn A., Apaijai N., Chattipakorn N. (2012). Effects of curcuminoids on frequency of acute myocardial infarction after coronary artery bypass grafting. Am. J. Cardiol..

[251] Shi Y., Zhang H., Huang S., Yin L., Wang F., Luo P., Huang H. (2022). Epigenetic regulation in cardiovascular disease: Mechanisms and advances in clinical trials. Signal Transduct. Target. Ther..

[252] Wu Y.-L., Lin Z.-J., Li C.-C., Lin X., Shan S.-K., Guo B., Zheng M.-H., Li F., Yuan L.-Q., Li Z.-h. (2023). Epigenetic regulation in metabolic diseases: Mechanisms and advances in clinical study. Signal Transduct. Target. Ther..

[253] Sarno F., Benincasa G., List M., Barabasi A.-L., Baumbach J., Ciardiello F., Filetti S., Glass K., Loscalzo J., Marchese C. (2021). Clinical epigenetics settings for cancer and cardiovascular diseases: Real-life applications of network medicine at the bedside. Clin. Epigenet..

